# Light at night and risk of breast cancer: a systematic review and dose–response meta-analysis

**DOI:** 10.1186/s12942-021-00297-7

**Published:** 2021-10-16

**Authors:** Teresa Urbano, Marco Vinceti, Lauren A. Wise, Tommaso Filippini

**Affiliations:** 1 CREAGEN - Environmental, Genetic and Nutritional Epidemiology Research Center, Department of Biomedical, Metabolic and Neural Sciences, University of Modena and Reggio Emilia, Modena, Italy; 2 Department of Epidemiology, Boston University School of Public Health, Boston, MA USA

**Keywords:** Breast cancer, Light-at-night, Lighting, Menopausal status, Dose–response meta-analysis

## Abstract

Breast cancer is the most common malignancy in women and the second leading cause of cancer death overall. Besides genetic, reproductive, and hormonal factors involved in disease onset and progression, greater attention has focused recently on the etiologic role of environmental factors, including exposure to artificial lighting such as light-at-night (LAN). We investigated the extent to which LAN, including outdoor and indoor exposure, affects breast cancer risk. We performed a systematic review of epidemiological evidence on the association between LAN exposure and breast cancer risk, using a dose–response meta-analysis to examine the shape of the relation. We retrieved 17 eligible studies through September 13, 2021, including ten cohort and seven case–control studies. In the analysis comparing highest versus lowest LAN exposure, we found a positive association between exposure and disease risk (risk ratio [RR] 1.11, 95% confidence interval-CI 1.07–1.15), with comparable associations in case–control studies (RR 1.14, 95% CI 0.98–1.34) and cohort studies (RR 1.10, 95% CI 1.06–1.15). In stratified analyses, risk was similar for outdoor and indoor LAN exposure, while slightly stronger risks were observed for premenopausal women (premenopausal: RR 1.16, 95% CI 1.04–1.28; postmenopausal: 1.07, 95% CI 1.02–1.13) and for women with estrogen receptor (ER) positive breast cancer (ER + : RR 1.09, 95% CI 1.02–1.17; ER–: RR 1.07, 95% CI 0.92–1.23). The dose–response meta-analysis, performed only in studies investigating outdoor LAN using comparable exposure assessment, showed a linear relation up to 40 nW/cm^2^/sr after which the curve flattened, especially among premenopausal women. This first assessment of the dose–response relation between LAN and breast cancer supports a positive association in selected subgroups, particularly in premenopausal women.

**Supplementary Information:**

The online version contains supplementary material available at 10.1186/s12942-021-00297-7.

## Introduction

Breast cancer is the most common cancer in women in both developed and undeveloped countries [1]. In Italy, there were an estimated 55,000 new cases in 2020 [2], and while incidence is increasing, mortality rates have significantly decreased across the years. Several factors appear to be involved in both etiology and prognosis of this malignancy, including selected genes, ageing, family history, reproductive factors, long-term use of postmenopausal female hormones, lifestyle [3, 4], and environmental factors such as exposure to chemical endocrine disruptors [5–9].

In 2007, the International Agency for Research on Cancer (IARC) defined night-shift work as ‘probably carcinogenic to humans’ i.e. belonging to category 2A [10], due to a possible link with breast cancer [11, 12] and with prostate and colorectal cancer [13]. The definition of night-shift work, also identified as graveyard shift, refers to a work schedule involving the sleeping hours of the general population. Among the exposure linked to graveyard shift work there is light-at-night (LAN), which induces disruption of the circadian rhythm and oxidative stress [14]. In particular, LAN could be involved in breast cancer etiology through different mechanistic pathways including DNA damage, impairment of melatonin and estrogen secretion, inflammation, and disruption of metabolic function [15]. Exposure to LAN can cause circadian and sleep disruptions, which may adversely affect different inflammatory and immunological pathways, thereby decreasing production of circulating natural killer cells or enhancing pro-inflammatory effects [16, 17]. Since sleep has antioxidant effects, its disruption may also cause increased production of reactive oxygen species [18, 19]. When evaluating exposure to LAN, both outdoor (e.g., streetlamps, illuminated buildings, lights from vehicles) and indoor artificial sources (e.g., domestic lights, electric devices-derived illumination) are relevant in increasing circadian disruption and therefore the risk of developing cancer. Outdoor LAN is generally assessed using satellite-derived data, while indoor is often evaluated through surveys on night habits and bedroom light characteristics [20].

Two recent meta-analyses summarized data on the association between LAN and breast cancer risk, investigating the effects of the highest vs. the lowest LAN exposure categories [21, 22]. Since the publication of these meta-analyses, three large studies of the LAN-breast cancer association have been published [23–25]. In the present report, we update the meta-analysis with these new studies, perform subgroup analyses by breast cancer subtype and other factors, and more comprehensively assess the epidemiological evidence about LAN and breast cancer risk. In addition, we apply dose–response meta-analyses to assess, for the first time, the shape of the association between LAN and breast cancer.

## Methods

We followed the Preferred Reporting Items for Systematic Reviews and Meta-Analysis (PRISMA) 2020 statement [26] to perform this review.

### Study identification and selection

The research question was configured according to PECOS statement (Population, Exposure, Comparator(s), Outcomes, and Study design): “Is exposure to light-at-night, as assessed through indoor and outdoor exposure to lighting sources, positively associated with risk of breast cancer in non-experimental studies?” and “Is there a dose–response association between LAN and breast cancer incidence?” [27]. Accordingly, we carried out a systematic literature search for publications available as of September 13, 2021 in the PubMed/MEDLINE, Embase and Web of Science (WoS) databases. We used search terms linked to “breast cancer” and “lighting” in PubMed, WoS, and Embase databases with no language restrictions (Additional file 1: Table S1). We also performed citation chasing by scanning the reference list of included studies and of previous reviews, as well as backward/forward citation retrieval to identify additional relevant papers [28]. Inclusion criteria were as follows: titles including LAN and breast cancer; mentioning case–control/case-cohort/cohort studies; monitoring LAN from space according to the US Defense Meteorological Satellite Program (DMSP) Operational Linescan System or from the Visible Infrared Imaging Radiometer Suite Day-Night Band (DNB); evaluating indoor LAN based on self-reported questionnaires and mentioning LAN levels as low, medium or high, and darkness and nightlight levels, and habits of sleeping with lights on; reporting of risk estimates for breast cancer, along with their 95% confidence intervals, or availability of enough data to calculate them.

Two authors reviewed all titles and abstracts independently, and any conflicts were resolved with the help of third author. For each included study, we extracted information about design, population size and characteristics, country, study period and years of follow-up, risk estimates (either odds ratio, risk ratio, or hazard ratio) along with their 95% confidence interval (CI) of breast cancer, adjustment factors, type of exposure assessment, and dose of exposure.

### Quality assessment

We assessed the quality in the included studies by using the Risk Of Bias In Non-randomized Studies of Exposures (ROBINS-E) tool [29]. We classified studies as having low, moderate, or high risk of bias according to seven domains: bias due to confounding; bias in selecting participants in the study; bias in exposure classification; bias due to departures from intended exposures; bias due to missing data; bias in outcome measurement; and bias in the selection of reported results. In Additional file 1: Table S2, we report criteria for risk of bias evaluation, performed by two authors. In case of disagreement, a third author helped in the final decision. A study’s overall risk of bias was considered high or moderate if at least one domain was judged at high or moderate risk, otherwise it was classified as having a low risk of bias.

### Statistical analysis

We performed a meta-analysis comparing breast cancer incidence in the highest versus lowest levels of LAN exposure using a restricted maximum likelihood random effect model, which bases estimates on a likelihood function calculated from a transformed set of data [30]. Additionally, whenever possible, we carried out a dose–response meta-analysis of breast cancer risk according to increasing LAN exposure through a random-effects model, using a one-stage approach as previously implemented in other fields [31–33]. Specifically, for each LAN category, we used the mean or the median value, or the midpoint for the intermediate categories, whichever was available. For the highest and lowest exposure categories, if the average values were not reported and were ‘open’, we used as boundary a value 20% higher or lower than the closest cut-point. We used a restricted cubic spline model with three knots at fixed percentiles (10th, 50th, and 90th) and we considered the correlation within each set of published effect estimates using generalized least-squares regression through a multivariate random-effect meta-analysis, incorporating the restricted maximum likelihood method [30, 34].

Besides the overall group, we also performed stratified analyses according to menopausal status (pre and postmenopausal), body mass index-BMI (< 25 and ≥ 25), estrogen receptor-ER status (ER + and ER–) of cases, and type of LAN exposure (outdoor and indoor). Furthermore, we explored the role of possible effect modifiers, by dividing the studies according to the country-specific estimated annual sunshine hours [35] into the three subgroups (< 2000, 2000–3000, and > 3000 annual mean sunshine hours), and country solar ultraviolet B (UV-B) radiation [36].

We assessed the potential for small-study bias using funnel plots for studies reporting highest versus lowest exposure, and by performing Egger’s test [37, 38] and trim-and-fill analysis [39]. We also evaluated the effect of variation across studies through the graphical overlay of study-specific predicted curves by using fixed and random effects [34]. Finally, we assessed heterogeneity by reporting I^2^ statistics, and by carrying out stratified analyses whenever possible such as for LAN exposure assessment method, menopausal status, participants’ weight (normal vs overweight/obese), and ER status. We used Stata software (v 16.1, 2021—Stata Corp., College Station, TX), namely its ‘meta’ and ‘drmeta’ routines, for data analysis.

## Results

**Fig. 1:**
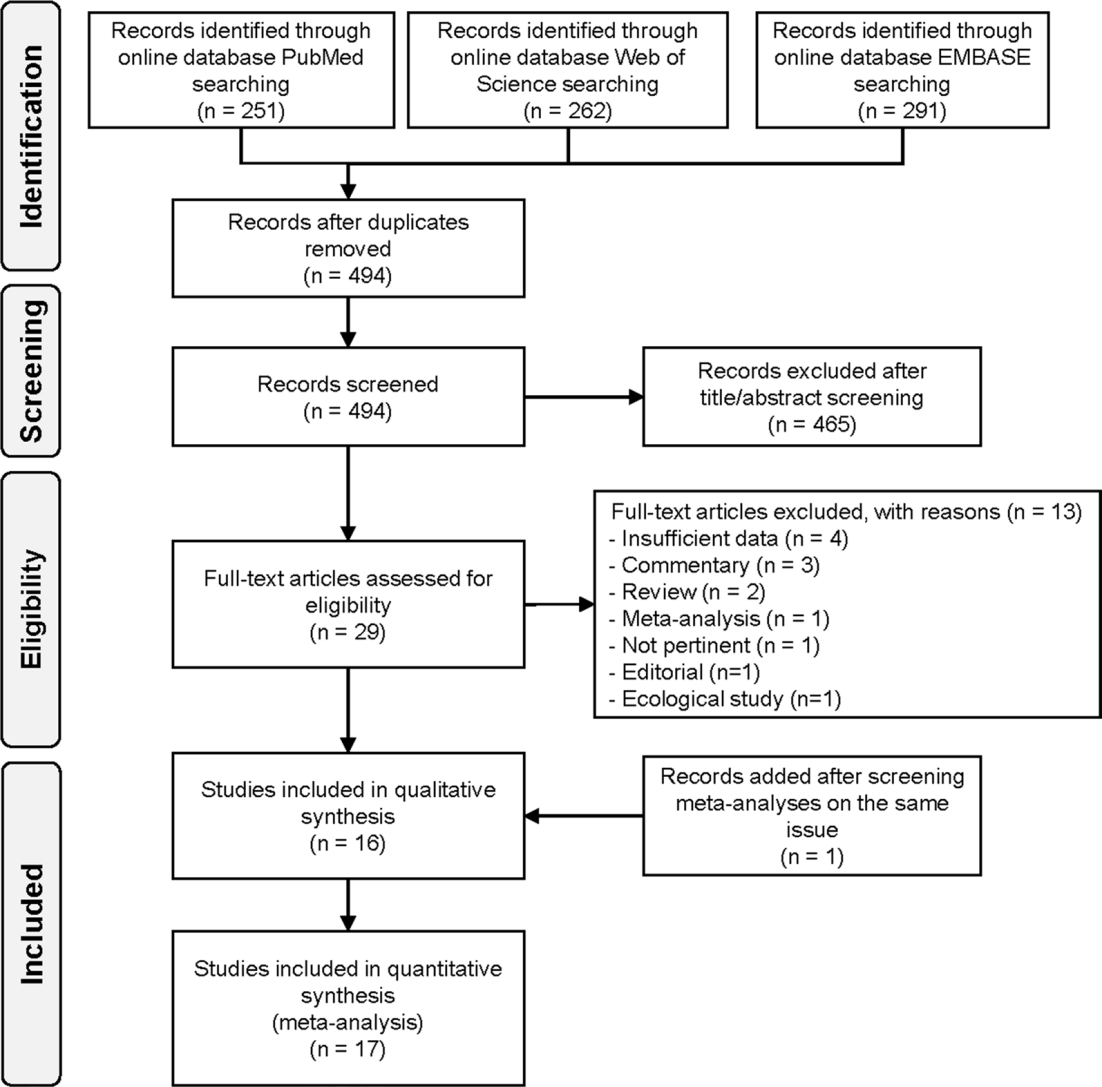
Flowchart summarizing the literature search and identification

Overall, of the 494 individual studies identified after removal of duplicates, we excluded 465 studies due to title and abstract screening, and 13 additional studies after full-text evaluation, leaving 17 studies eventually fulfilling inclusion criteria (Fig. 1). Main reasons of exclusion were the following: insufficient data, commentaries, reviews or meta-analyses, editorials, ecological studies, or lack of LAN exposure assessment (reasons reported in detail in Additional file 1: Table S3).

Table 1 presents characteristics of the 17 included studies, three with case-cohort design [40–42], six cohort [23–25, 43–45], and eight case–control [46–53]. The studies were published during 2001–2021, mainly in North America (N = 10), followed by Europe (N = 3), Israel (N = 2), Australia (N = 1), and China (N = 1). Follow-up duration was reported in only two studies (16 and 6.1 years) [23, 42]. LAN was assessed according to two main methods: from outdoor (N = 7) [23, 24, 40, 41, 43, 44, 52] and indoor (N = 11) sources [41–43, 45–51, 53]. In all cohort and case-cohort studies assessing outdoor LAN, the unit of measurement for LAN was nano-Watt/square centimeters/steradian (nW/cm^2^/sr) [23, 24, 40, 43, 44, 52]. The only exception was a Spanish study that assessed outdoor LAN using an index of outdoor blue light spectrum to calculate melatonin suppression index (MSI). MSI was estimated at each pixel of images of Madrid and Barcelona detected from the International Space Station (ISS) [41]. Indoor LAN data were evaluated based on self-reported questionnaires, for example, referring to use of lamps during the night or other sources of artificial light in the bedroom while sleeping. Additional characteristics of studies included in the systematic review are shown in the Additional file 1: Table S4.

**Table 1:** Characteristics of studies included in the systematic review

First author, year	Region	Cohort name	Study design, cohort years	Follow-up (years)	N cases /population	LAN assessment	Exposure by categories	Annual sunshine hours	UVB (Watt/meter^2^)	Cancer risk in all women	Matching and adjustment factors
Bauer 2013 [40]	US	Georgia Comprehensive Cancer Registry	Case-cohort, 2000–2007	NA	34053/61129	Outdoor (nW/cm^2^/sr) Satellite imagery for 1992–2007 constructed by the DMSP by averaging daily readings of the satellite sensors and removing cloud cover	T1: < 21 (10)	2000–3000	1.15–1.73	Ref 1.00	Race, tumor grade and stage, year of diagnosis, age at cancer diagnosis, MSA status, births per 1000 women aged 15–50, MSA population mobility, population over 16 in the labor force, and prevalence of cigarette smoking
							T2: 21–40 (31)			1.06 (0.97–1.16)	
							T3: > 41 (48)			1.12 (1.04–1.20)	
Clarke 2021 [24]	Denmark	Danish Nurse Cohort	Cohort	1993/1999–2012	745/16941	Outdoor (nW/cm^2^/sr) Data on annual residential outdoor LAN was obtained from the DMSP. High dynamic range data were used (available in 1996, 1999, 2000, 2003, 2004, 2006, and 2010). Calculation of annual exposure estimates: comparability across years and satellites was ensured by using the Interannual calibration coefficients provided by NOAA	T1: 0.00–21.9	< 2000	0.00–0.58	Ref 1.00	Age, calendar year, and entry at baseline, birth cohort, urbanicity, alcohol consumption, marital status, and night shift work, road traffic noise
							T2: 22.0–65.7			1.09 (0.90–1.31)	
							T3: 65.8–446.4			0.97 (0.77–1.23)	
Davis 2001 [46]	US	Cancer Surveillance System of the Fred Hutchinson Cancer Research Centre	Case–control, 1992–1995	NA	813/1606	Indoor Subjects were asked to classify the typical bedroom ambient light level according to the following six levels of darkness: 1) t h e subject wore a m ask to keep out light; 2) she could not see her hand in front of her face; 3) she could see to the end of her b ed; 4) she could see across the room; 5) she could barely read; an d 6) she could read comfortably	Q1: 0	2000–3000	1.15–1.73	Ref 1.00	Matched by age. Adjusted for parity, family history of breast cancer, oral contraceptive use, and recent discontinued use of hormone replacement therapy
							Q2: 0.00–0.4			1.00 (0.70–1.40)	
							Q3: 0.4–0.9			0.90 (0.60–1.20)	
							Q4: 0.9–2.9			1.00 (0.70–1.40)	
							Q5: > 2.9			1.00 (0.70–1.40)	
Fritschi 2013 [47]	Western Australia	Western Australia Cancer Registry	Population-based case–control , 2009–2011	NA	1205/2994	Indoor LAN assessed by asking women whether they could read easily at night at work (high exposure or could see but not well enough to read at work (medium exposure). Those women who did not fit either of these definitions, but had enough light to read in their bedroom when sleeping during the day were assigned low exposure	T1: lowest	2000–3000	1.15–1.73	Ref 1.00	Age
							T2: medium			1.06 (0.82–1.37)	
							T3: highest			1.25 (0.98–1.59)	
Garcia-Saenz 2018 [41 ]	Spain	MCC-Spain multicase-control study	Case-cohort, 2008–2013	NA	1219/2604	Outdoor Evaluation through MSI highly linked to blue light spectrum and melatonin suppression action spectrum	T1: lowest	> 3000	0.58–1.15	Ref 1.00	Age, center, educational level, socioeconomic status, UVI, BMI, tobacco, family history of breast cancer, chronotype, menopausal status, mutual adjustment for other light exposures
							T2: medium			0.91 (0.62–1.32)	
							T3: highest			1.47 (1.00–2.17)	
						Indoor Evaluated through the MCC-Spain questionnaire where it was defined as the level of light in the bedroom during sleeping time when the participants were at 40y of age or at the age of diagnosis or interview	Total darkness			Ref 1.00	
							Almost dark			0.73 (0.44–1.21)	
							Quite illuminated			0.77 (0.39–1.51)	
							Dim light			1.01 (0.60–1.69)	
Hurley 2014 [43]	US	California Teachers’ cohort study	Cohort, 1995–2010	NA	106 731 (tot)	Outdoor (nW/cm^2^/sr) Data derived from satellite imagery data obtained from the archive of the US DMSP Operational Linescan System, maintained by NOAA’s Earth Observation Group	Q1: 0–14.2 (7.1)	2000–3000	1.15–1.73	Ref 1.00	Age strata (1 year), race/birthplace, family history of breast cancer, age at menarche, pregnancy history, breastfeeding history, physical activity, BMI, alcohol consumption, menopausal status and hormone therapy use combination, smoking status, smoking pack-years, neighborhood socio-economic status, and urbanization
							Q2: 14.3–26.4 (20.35)			1.05 (0.95–1.16)	
							Q3: 26.5–37.4 (31.95)			1.06 (0.95–1.17)	
							Q4: 37.5–53.3 (45.4)			1.05 (0.95–1.17)	
							Q5: 53.4–175.2 (114.3)			1.12 (1.00–1.26)	
						Indoor Indicators were based on responses to the questions: “During the past year, have you used a bright light at night while sleeping at home?	Non-user			Ref 1.00	
							Light			1.17 (0.87–1.57)	
							Medium			0.99 (0.82–1.20)	
							Heavy			1.13 (0.84–1.52)	
James 2017 [44]	US	Nurses’ Health Study II	Cohort, 1989–2013	NA	3549/109672	Outdoor (nW/cm^2^/sr) DMSP Global Radiance Calibrated Night-time Lights high-dynamic range data Interannual calibration coefficients were provided by NOAA to derive exposure estimates	Q1: 0.4–7.2 (4.3)	2000–3000	1.15–1.73	Ref 1.00	Stratified for age at follow-up and calendar year. Adjusted for benign breast disease history, family history of breast cancer, age at menarche, parity, and age at first birth, height, white race, BMI, BMI at 18, oral contraceptive use, mammography screening, menopausal status, smoking, status, alternative healthy eating index, physical activity, marital status, living alone, personal income, shift work after 1989, region, PM2.5, census-tract median home value, income, and population density
							Q2: 6.3–15.9 (12.4)			1.05 (0.94–1.18)	
							Q3: 14.4–30.1 (22.9)			1.01 (0.90–1.13)	
							Q4: 26–53.3 (37.2)			1.08 (0.97–1.22)	
							Q5: 41.4–248.1 (64.0)			1.14 (1.01–1.29)	
Johns 2018 [42]	UK	Generations Study	Case-cohort, 2003–2012	6.1 years (mean)	1775/105866	Indoor Women were asked to report their level of exposure to LAN over the year prior to recruitment and at age 20 years, in the room in which they slept, in the categories; ‘light enough to read’; ‘light enough to see across the room, but not read’; ‘light enough to see your hand in front of you, but not to see across the room’; and ‘too dark to see your hand, or you wear a mask’	T1: low	< 2000	0.00–0.58	Ref 1.00	Year of birth, history of benign breast disease, breast cancer in a first-degree relative, socioeconomic score, age at menarche, age at first birth, parity, duration of breastfeeding, oral contraceptive use, hormone replacement therapy use, menopausal status, and age at menopause where applicable, premenopausal, and postmenopausal BMI, alcohol consumption, smoking and physical activity level
							T2: medium			1.00 (0.89–1.12)	
							T3: high			1.01 (0.88–1.15)	
Keshet-Sitton 2016 [48]	Israel	Various Medical Centers in Tiberius	Case–control, 2010–2014	NA	95/278	Indoor Sleep quality, falling asleep with TV on (turning the TV off before sleeping), sleeping with the TV on for most of the night, exposure to outdoor and indoor lighting in the sleeping habitat, use of bed lamps or room lamps for reading before retiring to sleep were variables that were part of the questionnaire. Answers were scaled from 1 to 5, where 1 was lowest and 5 highest	Reading with bed light illumination	> 3000	1.15–1.73	0.82 (0.68–0.99)	Matched by age and residential area
							Sleeping with closed shutters			0.81 (0.67–0.97)	
							Resides near strong ALAN sources			1.52 (1.1–2.12)	
Kloog 2011 [49]	Northern Israel	Breast cancer in Norther Israel study	Case–control, 2000–2008	NA	794/1679	Indoor Nighttime bedroom-light level was evaluated using a 4-point scale: from completely dark (score of 1) to strong light (score of 4). The exact wording of the question was as follows: “How do you define your nighttime bedroom-light level? “1” (completely dark), “2” (low light), “3” (average light), or “4” (very strong light—all lights switched on). Other LAN exposure-related questions included the availability of bedroom shutters and sleeping with the television left on. The answers to these questions were coded dichotomously, that is, yes or no	TV off while sleeping	> 3000	1.15–1.73	Ref 1.00	Matched by age, location of primary clinic, and ethnicity. Adjusted for family history of cancer, parity, oral-contraceptive use, and hormone-replacement therapy
							TV on while sleeping			0.91 (0.73–1.15)	
							Bedroom light (scoring 1 to 4)			1.22 (1.12–1.31)	
Li 2010 [50]	US	NA	Case–control, NA	NA	363/719	Indoor Questions targeted detailed information on the participants’ sleeping patterns and bedroom light environment in the 10 years prior to diagnosis (or reference date for controls). Questions on sleeping patterns and bedroom light environment included: (1) whether hours slept occurred during the daytime or at night; (2) whether the lights were kept on while sleeping; (3) the presence of streetlights, lighted signs, or any other exterior light which affected the sleeping area; (4) pulling a window shade or curtain while sleeping; (5) having radio, TV, and/or hall lighting on while sleeping, and (6) hours of sleep per day	Keeping light off while sleeping	2000–3000	1.15–1.73	NA	Age group, race, BMI, age at first menstrual period, family history of breast cancer, age at first full-term birth, months of lifetime breast feeding, cigarette smoking, and alcohol drinking
							Keeping light on while sleeping				
							No streetlights, lighted signs, or any other exterior light outside sleeping area				
							Streetlights, lighted signs, or any other exterior light outside sleeping area				
							Clock radio, TV, hall light, etc. off while sleeping				
							Clock radio, TV, hall light, etc. on while sleeping				
O' Leary 2006 [51]	US	Long Island Breast Cancer Study Project	Case–control, 1996–1997	NA	576/1161	Indoor Residential light-at-night exposures were ascertained for the 5-year period prior to the reference date compared with the distant past. Questions covered sleep hours, frequency of turning on lights during sleep hours, and length of time the light was on	Q1	2000–3000	1.15–1.73	Ref 1.00	Age at reference date, parity, family history (defined as a mother, sister, or daughter with breast cancer), education (defined as less than high school or high school graduate as the referent vs. some college, college graduate, and post college education), and history of benign breast disease
							Q2			0.98 (0.66–1.44)	
							Q3			0.71 (0.43–1.16)	
							Q4			0.99 (0.67–1.48)	
							Q5			1.12 (0.80–1.57)	
Ritonja 2020 [52]	Canada	NA	Population-based case–control , 2005–2010	NA	844/1749	Outdoor DNB (nW/cm^2^/sr) For DNB, 2012 was chosen because it was the earliest year for which these data are available. Sensitivity to light in the wavelength range 500–900 nm	T1: 0.00–22.07 (11.035)	< 2000	0.00–0.58	Ref 1.00	Age, ethnicity, menopausal status, family history of breast cancer, age at menarche, BMI, household income, education, parity, and age at first birth, years of oral contraceptive use, age at first mammography screening, smoking status, night work status, average alcoholic drinks/week, average population density, and average neighborhood income
							T2: 22.08–32.79 (27.435)			0.97 (0.75–1.26)	
							T3: 32.83–149.47 (91.15)			0.95 (0.70–1.27)	
						Outdoor DMSP (nW/c m^2^/sr) The DM SP data are fro m the annual “ra diometrica lly calibrate d” product for 2010. Sensitivity to light in the wavel ength range 500–900 nm	T1: 0.00–123.05 (61.525)			Ref 1.00	
							T2: 123.10–194.62 (158.86)			1.05 (0.82–1.34)	
							T3: 194.75–628.56 (411.655)			0.83 (0.63–1.09)	
White 2017 [45]	US and Puerto Rico	Sister Study	Cohort, 2003–2009	3 years	50533	Indoor Women were asked detailed questions about any types of light present while sleeping, frequency of waking up at night, frequency of naps and sleep medication use	Q1: No light	2000–3000	1.15–1.73	Ref 1.00	Age, race, education, income, marital status, postmenopausal hormone use, use or oral contraceptives, alcohol consumption, age at menarche, parity, age at first birth, age at menopause, pack years of smoking and physical activity
							Q2: Daylight			0.87 (0.66–1.15)	
							Q3: Nightlight			0.97 (0.87–1.08)	
							Q4: Light outside the room			1.01 (0.90–1.13)	
							Q5: Light/television in room			1.09 (0.93–1.26)	
Xiao 2020 [23]	US	NIH-AARP Diet and Health study	Cohort, 1995–2005	16 years	186 981 (tot)	Outdoor (nW/cm^2^/sr) DMSP Global Radiance Calibrated Night-time Lights high-dynamic range data	Q1: 2.4–6.3 (4.3)	2000–3000	1.15–1.73	NA	Age, state of residence, ethnic, education, marital status, breast cancer in first-degree relatives, age of menarche, age at first childbirth, contraceptive use, age at menopause, menopause hormonal therapy, smoking status, vigorous physical activity, alcohol consumption, number of breast biopsies, mammogram in the past 3 years, healthy eating index-2005, census tract median home value, poverty rate and population density
							Q2: 10.8–16.1 (13.3)				
							Q3: 23–31 (26.9)				
							Q4: 40.6–51.5 (45.7)				
							Q5: 66.6–90.3 (76.6)				
Xiao 2021 [25]	US	Southern Community Cohort Study	Cohor t	NA	1224/43 500	Outdoor (nW/cm^2^/sr) U.S. Defense Meteorological Satellite Program’s Operational Linescan System with high-dynamic range data to avoid saturation in high-LAN areas	Q1: 0.8–1.7 (1.2)	2000–3000	1.15–1.73	Ref 1.00	Age, education, marital status, income, health insurance coverage, smoking, family history of breast or ovarian cancer among first-degree female relatives, mammogram, age at menarche, postmenopausal status, ever use of menopausal hormone therapy, average number of alcoholic drinks consumed per day, and population density and percentage of households living under the 2000 federal poverty line in the census tract
							Q2: 3.8–9.1 (6.2)			0.98 (0.82–1.18)	
							Q3: 16.3–24.3 (20.3)			1.13 (0.93–1.36)	
							Q4: 32.3–39.5 (35.9)			1.01 (0.82–1.25)	
							Q5: 48.9–68.2 (55.6)			1.27 (1.00–1.60)	
Yang 2019 [53]	China	Jiujiang breast cancer study	Population-based case–control , 2013–2016	NA	401/802	Indoor Validated 17-item SFQ to comprehensively collect 5-year sleep habits before their BC diagnosis for cases and the most recent 5-year sleep habits for population controls. The SFQ includes eight subscales: habitual bedtim e information (four items), self-evaluation of sleep quality (one item), insomnia frequency (one item), history of sleep medication use (three items), LAN exposure (two items), frequency of nighttime waking (one item), nightshift work (four items), and habitual nap time (one item)	Level 1 (wore a mask to keep out light or could not see hand in front of face)	2000–3000	1.15–1.73	Ref 1.00	Age, education, family income, occupation, menopausal status, number of live births, use of menopausal hormones, age at menarche, age at first birth, marital status, family history of breast cancer, smoking, alcohol drinking, fruit and vegetable consumption, regular physical activity, BMI, and adjusted mutually for other sleep variables including sleep duration, sleep quality, light exposure at night, night/shift work, and sleep medication use
							Level 2 (could see only the hazy outline of the bedroom)			1.08 (0.75–1.93)	
							Level 3 (could barely read)			1.1 (1.02–2.35)	
							Level 4 (could read comfortably)			1.19 (1.06–2.68)	

*ALAN* artificial light at night, *BMI* body mass index, *CI* confidence interval, *DMSP* US Defense Meteorological Satellite Program Operational Linescan System, *DNB* Visible Infrared Imaging Radiometer Suite Day-Night Band, *MSA* metropolitan statistical area, *MSI* melatonin suppression index, *NA* not assessed, *NIH–AARP* National Institutes of Health–American Association of Retired Persons, *NOAA* National Oceanic and Atmospheric Administration,*Ref* reference, *RR* risk ratio, *SFQ* sleep factors questionnaire, *UVI* ultraviolet index

Risk of bias assessment (Additional file 1: Table S5) showed that most studie s were at low risk of bias due to confounding, while four w ere at moderate risk of bias because they did not control for some breast cancer risk factors, typically family history of breast cancer, postmenopausal hormone use, or sm oking [24, 40, 47, 48]. Concerning exposure assessment, studies assessing outdoor LAN exposure were at low risk of bias [23–25, 40, 44, 52], while those assessing both outdoor and indoor LAN or only indoor LAN were generally at moderate risk due to possible misclassification bias [41–43, 45–47, 49–51, 53]. One study was considered at high risk of bias because exposure assessment was based on a non-validated self-administered questionnaire [48]. All other domains were considered at low risk of bias in all studies, although four studies were judged at moderate risk of bias because some information (i.e., smoking or menopausal status) had been collected but not reported, no differences were found, or no data were presented [44, 46, 51].

**Fig. 2:**
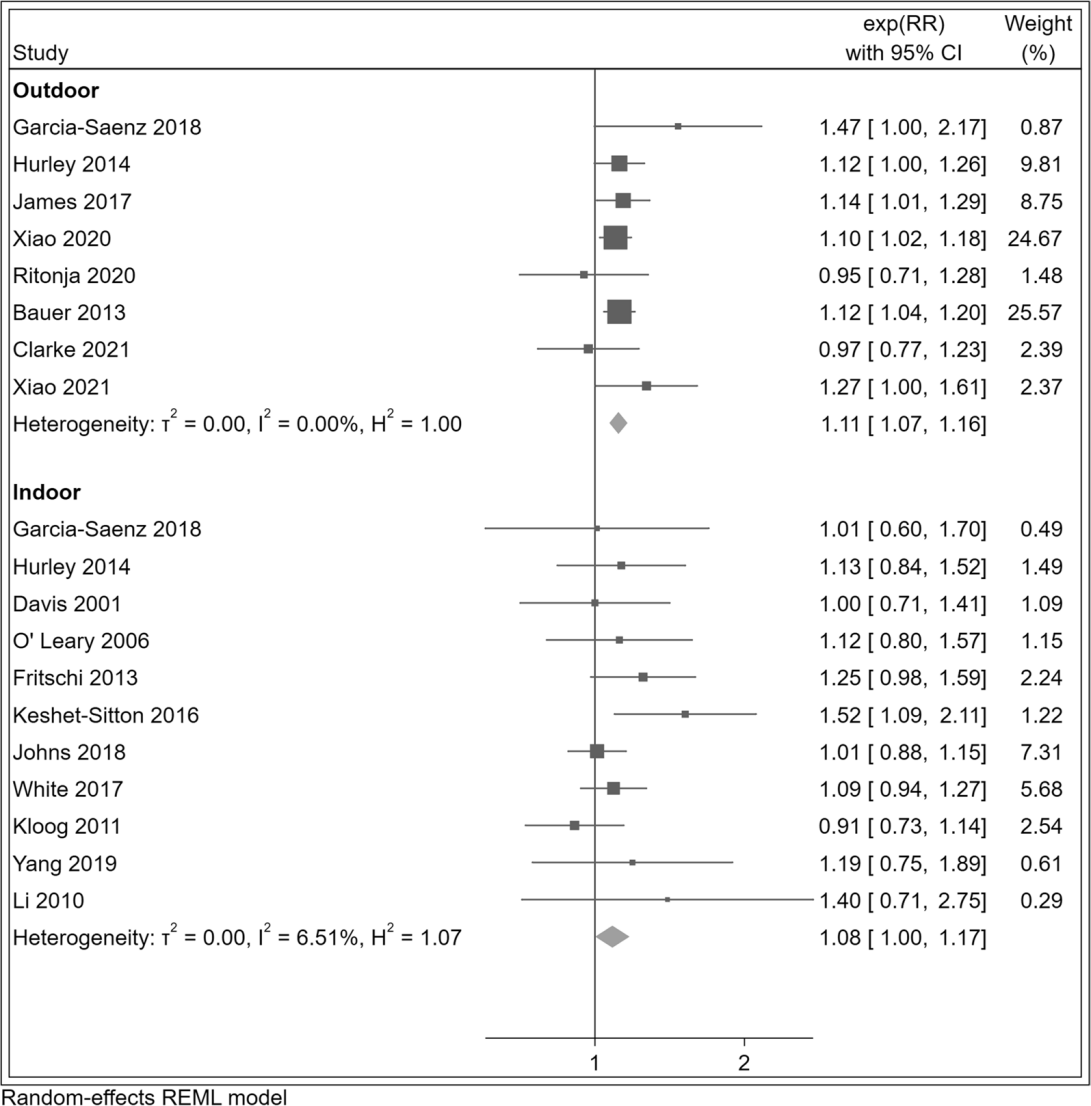
Risk ratio (RR) with 95% confidence interval (CI) for the association between light at night exposure and r isk o f breast cancer (N = 17 studies) com paring the highest versus the lowest exposure category in studies assessing outdoor and indoor exposure. The squares represent point estimates of RR and horizontal lines represent their 95% confidence intervals (CIs). The area of each square is proportional to the inverse of the variance of the estimated log RR. The diamonds represent the combined RR for each subgroup and the overall RR for all studies. The solid line represents RR = 1

Comparing the highest versus the lowest LAN exposure category, we consistently found positive associations with breast cancer risk (summary RR = 1.11, 95% CI 1.07–1.15). In subgroup analyses (Table 2), we found positive associations for outdoor (RR = 1.11, 95% CI 1.07–1.16) and indoor (RR = 1.08, 95% CI 1.00–1.17) LAN exposure, as well as both for case–control (RR = 1.11, 95% CI 0.97–1.28) and cohort studies (RR = 1.11, 95% CI 1.07–1.15) as shown in Fig. 2 and Additional file 1: Figure S1.

**Table 2:** Summary risk ratios (RRs) and 95% confidence interval (CI) for the association between breast cancer risk and light at night exposure comparing the highest versus the lowest exposure categories for overall study population, outdoor and indoor exposure with selected subgroups

Breast cancer	All studies			Outdoor			Indoor		
	***n***	RR (95% CI)	*I*^*2*^ (%)	*n*	RR (95% CI)	*I*^*2*^ (%)	*n*	RR (95% CI)	***I***^***2***^** (%)**
All women	17	1.11 (1.07–1.15)	0.0	7	1.11 (1.07–1.16)	0.0	11	1.08 (1.00–1.17)	6.5
Study design									
Cohort/case-cohort studies	9	1.11 (1.07–1.15)	0.0	8	1.11 (1.07–1.16)	0.0	4	1.05 (0.96–1.15)	0. 0
Case–control studies	8	1.11 (0.97–1.28)	31.5	1	0.95 (0.71–1.28)	-	7	1.14 (0.98–1.34)	34.2
Menopausal status									
Premenopausal	8	1.16 (1.04–1.28)	2.3	4	1.22 (1.08–1.39)	0.0	4	1.04 (0.88–1.23)	0.0
Postmenopausal	9	1.07 (1.02–1.13)	0.0	5	1.07 (1.00–1.14)	5.9	4	1.08 (0.95–1.23)	4.3
BMI									
< 25 kg/m^2^	2	1.17 (1.00–1.36)	38.9	2	1.17 (1.00–1.36)	39.0	-	–	-
≥ 25 kg/m^2^	2	1.07 (0.87–1.32)	53.8	2	1.07 (0.87–1.32)	53.8	-	–	–
Estrogen receptor status									
ER +	7	1.09 (1.02–1.17)	0.0	4	1.12 (0.95–1.32)	44.8	3	1.06 (0.95–1.18)	0.0
ER –	7	1.07 (0.92–1.23)	0.0	4	1.12 (0.92–1.35)	0.0	3	1.01 (0.81–1.25)	0.0
LAN/sunshine hours									
< 2000 h	3	0.99 (0.89–1.11)	0.0	2	0.96 (0.80–1.16)	0.0	1	1.01 (0.88–1.15)	–
2000–3000 h	11	1.12 (1.08–1.17)	0.0	5	1.12 (1.07–1.17)	0.0	7	1.13 (1.02–1.25)	0.0
> 3000 h	3	1.30 (1.11–1.52)	15.4	1	1.47 (1.00–2.17)	–	3	1.11 (0.79–1.56)	66.3
Equinoctial UVB									
0.0–0.58 W/m^2^	3	0.97 (0.87–1.08)	0 .0	2	0.91 (0.76–1.09)	0.0	1	1.01 (0.88–1.15)	–
0.58–1.15 W/m^2^	1	1.27 (0.89–1.82)	–	1	1.47 (1.00–2.17)	–	1	1.01 (0.60–1.70)	–
1.15–1.73 W/m^2^	13	1.15 (1.10–1.19)	5.6	5	1.12 (1.07–1.17)	0.0	9	1.20 (1.12–1.27)	0.0

*BMI* body mass index, *CI* confidence interval, *ER* estrogen receptor, *h.* hours, *I*^*2*^* (%)* heterogeneity, *LAN* l ight at night, *n* number of studies, *RR* risk ratio, *UVB* ultraviolet B

**Fig. 3:**
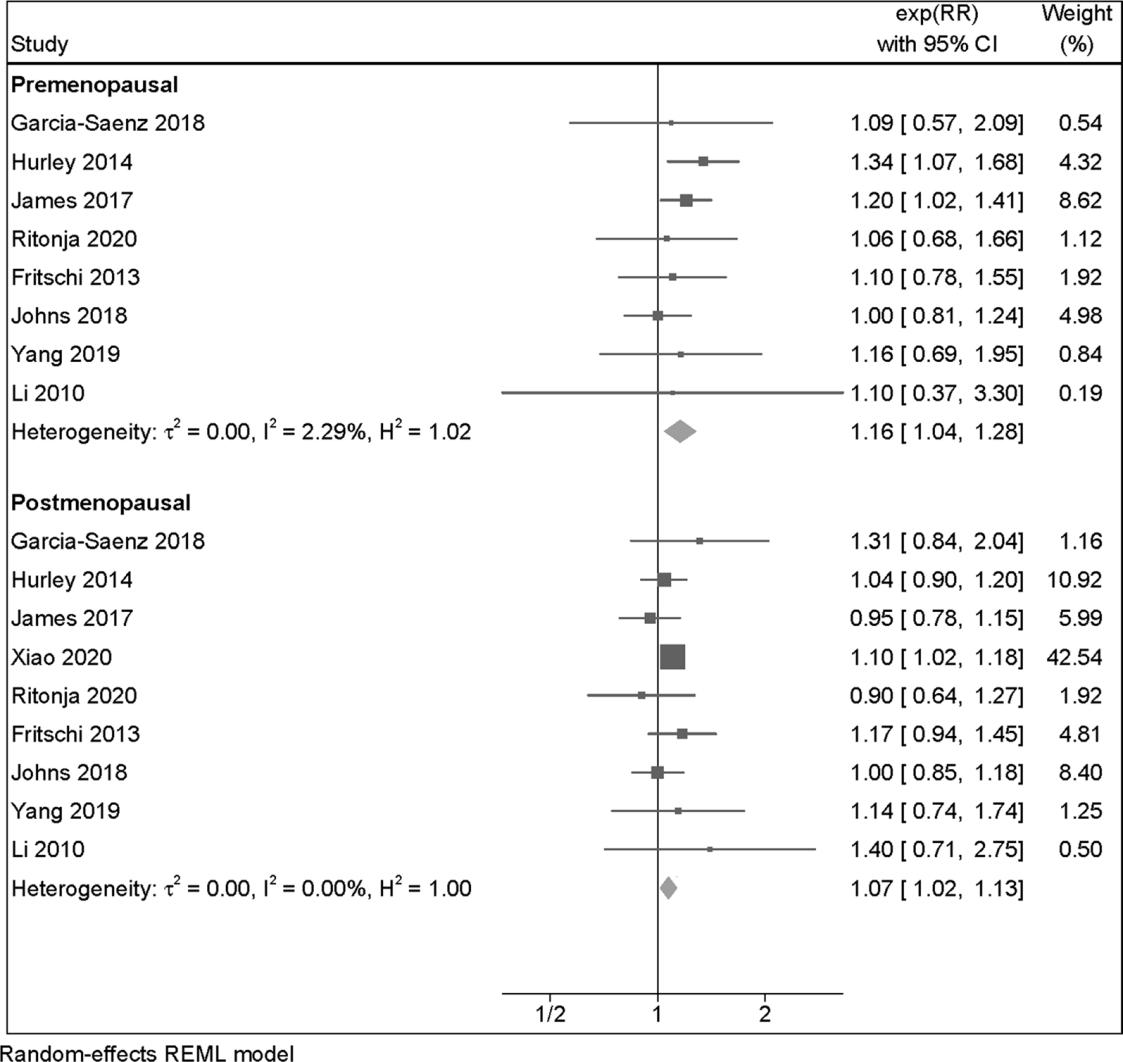
Risk ratio (RR) with 95% confidence interval (CI) for the association between light at night exposure and risk of breast cancer (N = 9 studies) among premenopausal and postmenopausal women, comparing the highest versus the lowest exposure category. The area of each grey square is proportional to the inverse of the variance of the estimated log RR. Black diamonds represent point estimates of RR and horizontal lines represent their 95% confidence intervals (CIs). The open diamonds represent the combined RR for each subgroup and the overall RR for all studies. The solid line represents RR = 1

Eight studies assessed breast cancer risk among both pre and postmenopausal women at the moment of diagnosis, while one was restricted to postmenopausal women only. The summary RR was slightly stronger among premenopausal women (RR = 1.16, 95% CI 1.04–1.28) than postmenopausal women (RR = 1.07, 95% CI 1.02–1.13) (Fig. 3). A slight positive association among premenopausal women also emerged in the cohort/case-cohort study subgroup and for outdoor LAN exposure. Conversely, for the case–control category and indoor LAN exposure, the RR was slightly higher among postmenopausal women (Table 2 and Additional file 1: Figure S2).

**Fig. 4:**
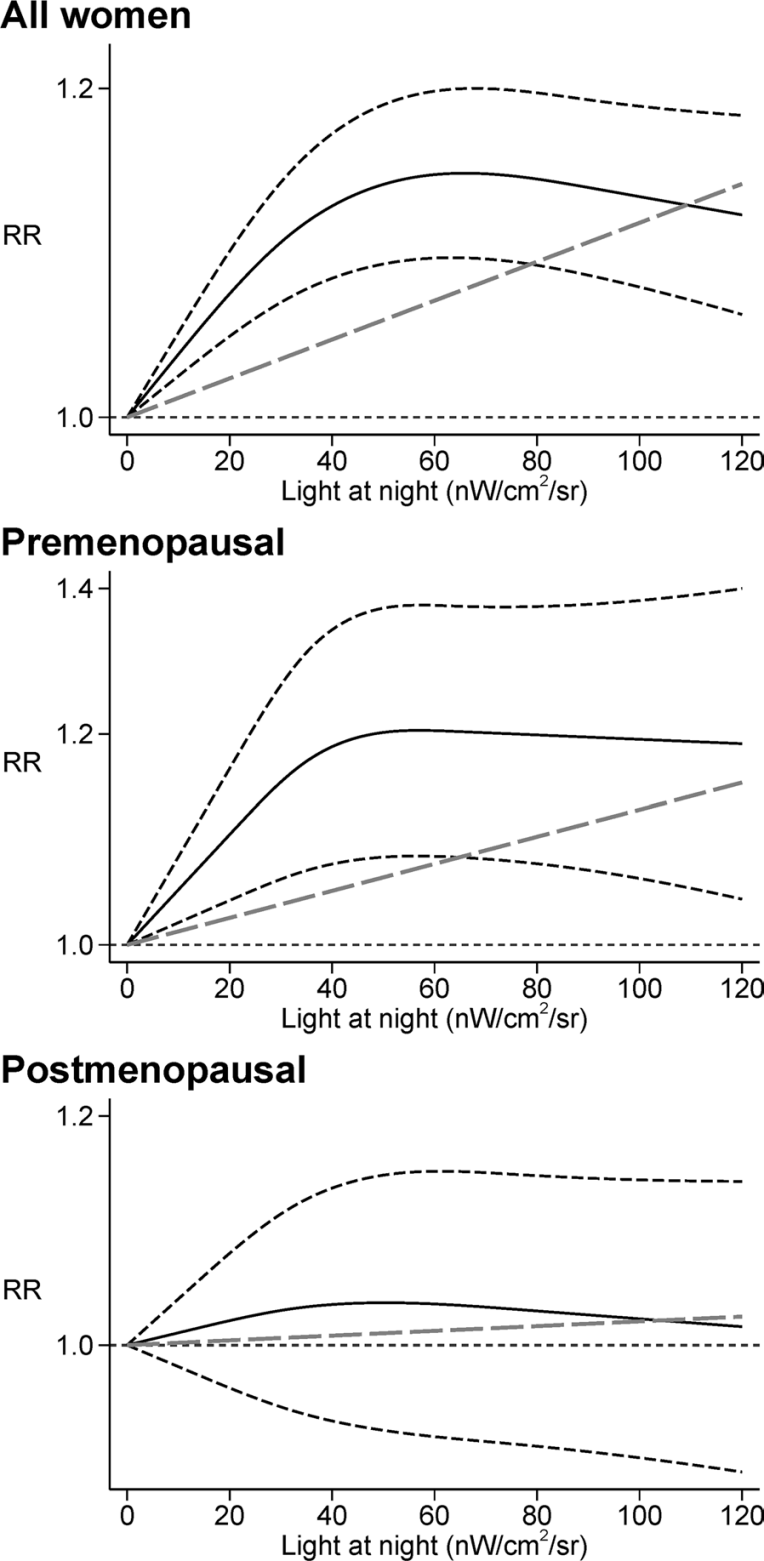
Dose–response meta-analysis between light at night and risk of breast cancer among all women [23–25, 40, 43, 44, 52] and between light at night and risk of breast cancer in premenopausal [43, 44, 52] and postmenopausal women [23, 43, 44, 52]. Spline curve (black solid line) with 95% confidence limits (black dashed lines), and linear trend estimation (long-dashed gray line). *RR* risk ratio

In the dose–response meta-analysis, we found a positive linear relation up to 40 nW/cm^2^/sr of outdoor LAN exposure, after which a plateau was reached (Fig. 4). A substantially comparable pattern was noted for all women and in analyses stratified according to menopausal status.

Concerning possible effect-modification by ER status, few differences emerged between women with ER + and E R–b r east cancer , for whom the summary RRs were 1.09 (95% CI 1.02–1.17) and 1.07 (95% CI 0.92–1.23), respectively (Table 2 and Additional file 1: Figure S3). In addit ion, in the dose–response meta-analysis we found that the risk was slightly higher in the ER  −  subgroup compared to the ER + one. A difference also emerged above 30 nW/cm^2^/sr of outdoor LAN exposure, when the curve flattened in the ER + subgroup while continued to increase in the ER − one (Additional file 1: Figure S4).

With regards to BMI status, the two studies of breast cancer risk among women with BMI < 25 or BMI ≥ 25 yielded similar positive summary RRs in both categories, though RRs were stronger in the normal-weight (BMI < 25) group (RR = 1.17, 95% CI 1.00–1.36 and RR = 1.07, 95% CI 0.87–1.32, respectively (Table 2 and Additional file 1: Figure S5). A monotonic positive association emerged in the dose–response meta-analysis for the two BMI subgroups, despite the very low number of studies (Additional file 1: Figure S6).

We also performed stratified analyses according to the annual sunshine hours’ map of the world (Table 2 and Additional file 1: Figure S7), dividing the 17 studies by country latitude. We divided the countries in three different groups of annual sunshine hours (< 2000 h: Canada, UK, Denmark; 2000–3000 h: US, Western Australia, China; > 3000 h: Spain, Israel). Countries with annual sunshine hours < 2000 exhibited null association (RR = 0.97, 95% CI 0.87–1.08). Conversely, we found a stronger association between LAN in the subgroup of countries with 2000 to 3000 annual sunshine hours (RR = 1.12, 95% CI 1.08–1.17) as well as in countries with more than 3000 annual sunshine hours, i.e., Spain and Israel (RR = 1.30, 95% CI 1.11–1.52) (Additional file 1: Figure S8). These findings were also observed in analyses stratified by postmenopausal status (Additional file 1: Figure S9) and indoor/outdoor exposure, although for indoor exposure, the risk ratio was slightly higher in countries with 2000 to 3000 annual sunshine hours than those with more than 3000 (Table 2 and Additional file 1: Figure S10). Finally, when we accounted for estimated equinoctial UV-B irradiance map (Additional file 1: Figure S11) we found a null association between LAN and breast cancer risk in studies from countries with less UV-B irradiance (RR = 0.97, 95% CI 0.87–1.08), while an inverse association was observed in the outdoor exposure subgroup (RR = 0.91, 95% CI 0.76–1.09). Conversely, there was a positive association in studies from countries with higher (> 0.58 W/m^2^) UV-B irradiance levels (Additional file 1: Figure S12), as also confirmed when considering either outdoor or indoor exposure (Table 2).

Exclusion of the one study [48] considered at high risk of bias did not substantially alter the results (Additional file 1: Table S6). Findings were also similar when we additionally excluded the two studies considered at moderate risk of bias in selection of reported results [46, 51] (Additional file 1: Table S7). To further limit the effect of potential biases, we then performed the analyses excluding three additional studies [24,40, 47] considered at moderate risk of bias due to confounding. Still, there were little changes in the results, and the estimates were substantially confirmed (Additional file 1: Table S8). Similarly, analysis of conditional study-specific lines arising from the estimated random-effects model yielded homogeneous results overall and among premenopausal women, while among postmenopausal women, slightly higher variation was noted (Additional file 1: Figure S13). Finally, evaluation of small-study bias suggested no occurrence of bias due to symmetric distribution and no studies were added when running trim-an-fill analysis both in overall studies (Additional file 1: Figure S14) and in analyses stratified by menopausal status (Additional file 1: Figure S15) and exposure assessment (Additional file 1: Figure S16).

## Discussion

Higher urbanization has prompted substantial changes in peoples’ lifestyles as compared with our ancestors. Nowadays, over 80% of the World’s population and close to 100% of the people in the United States and Europe live under skies polluted by light [54], one of the key environmental factors characterizing the Western world environment . Besides residence-related artificial light (i.e., urban light pollution), other sources of non-natural LAN are electronic devices (TVs, smartphones, tablets, computers, etc.) or lights turned on during night at home or at the workplace. The possible carcinogenic effects of LAN has been recently assessed also by the U.S. National Toxicology Program (NTP) cancer hazard assessment [55]. NTP concluded that there was moderate evidence for a causal relation between LAN exposure and human cancer, since LAN may act through different mechanisms of circadian disruption and its biological effects are the same of well-known recognized carcinogens [55].

Over the last twenty years, the association between LAN exposure and breast cancer risk has been assessed primarily in occupational settings, specifically among night-shift workers [56–60]. These studies generally found a slight to substantial excess for breast cancer in women working graveyard shifts. Most recently, epidemiological studies investigating LAN exposure, in most cases independently from nightshift work, and its association with risk of breast cancer in the general population have greatly increased. In longitudinal studies, metrics of outdoor LAN have been collected through sophisticated methods such as the US DMSP Operational Line-Scan System or the Visible Infrared Imaging Radiometer Suite DNB, and expressed as nW/cm^2^/sr, except for one study [41], which was based on a visual artificial light-at-night (ALAN) assessment to estimate ground-based spectrum of the light emission, and melatonin suppression index for outdoor blue light spectrum. A Canadian study used both DNB and DMSP data. Even if DNB has a higher resolution and a calibrated radiometer, DMSP was used in our analysis for comparison with other studies [52].

Most case–control studies, in turn, have assessed LAN exposure using self-administered questionnaires regarding sleep and/or night habits. Exposure assessment in these studies has included frequency of waking up and turning on lights during night, sleeping with the TV on or off, darkness level in the room, residency near strong artificial LAN sources, wearing a mask while sleeping, keeping lights on or off while sleeping.

There is some biological plausibility for a LAN breast cancer association, given the observation that repeated exposure to artificial light during night hours might induce DNA damage and oxidative stress, alter melatonin and estrogen synthesis and metabolism, inflammation and immune function, and disrupt metabolic function [18]. More specifically, three mechanisms have been proposed to explain the link between LAN and some types of cancers. LAN could inhibit melatonin secretion directly [61], through sleep deprivation (also affecting cell proliferation and cytokines production [62]), and through chronodisruption [63, 64]. Along these lines, previous studies referred more generally to night shift work than to LAN exposure, but night shift work is a far more complex exposure, including among other changes in sleep habits, sleep deprivation, eating during the night [61]. For this reason, we focused our attention more specifically on exposure to LAN as a factor associated with higher incidence of breast cancer, rather than night shift work, though the latter may confound to some extent the association between LAN and breast cancer risk.

We found a consistent positive association between LAN and breast cancer risk overall and among several subgroups, including premenopausal women, those with BMI < 25, and those living in countries experiencing more than 3000 sunshine hours a year. The risk of developing breast cancer was almost monotonically associated with outdoor LAN up to the value of 40 nW/cm^2^/sr, above which the threshold of the curve flattened. The association was stronger among premenopausal women, suggesting that younger women or women with higher endogenous levels of estrogens may have greater susceptibility to the effects of LAN. Effect measure modification by menopausal status could be due to different underlying biological mechanisms. Previous studies have reported that the suppressive effect of LAN on melatonin secretion may be stronger among younger people, tending to decrease with age [62, 65]. In addition, LAN may impact on the length of the menstrual cycle through endocrine-disrupting properties, thus leading to higher breast cancer risk in the premenopausal period [66].

With regard to confounding factors, solar UV-B radiation is thought to be protective for breast cancer development. The inverse association between cancer risk and UV-B radiation was hypothesized for the first time by the Garland brothers in 1980 [67] who theorized sunlight-induced increases in vitamin D_3_ may confer protection. The final product of the vitamin D_3_ metabolism is the calcitriol, which has many anti-carcinogenic properties including inhibiting cellular proliferation [68]. Across the years, many studies have investigated the potential protective role of the UV-B radiation against different types of cancer [69–72]. A remaining question is the relationship between LAN and ultraviolet radiation. We found a positive association between LAN and breast cancer risk in countries exposed to higher levels of UV-B radiation (> 0.58 W/m^2^). Conversely, there was no association between LAN and breast cancer risk in countries with low UV-B irradiance (< 0.58 W/m^2^).

We considered another confounding factor that also correlates with UV-B radiation and could influence the outcome risk: the influence of annual sunshine hours [73, 74]. We found an increased breast cancer risk associated with LAN in countries where annual sunshine exposure exceeded 3000 h. A positive association was also found in countries where sunshine ranged 2000–3000 h/years, while no appreciable association was observed in countries where there were fewer sunshine hours. This could be explained by different habits of people living in different countries, which may reflect epigenetics adaptation [75]. As indicated by a 2014 Italian study, humans’ biological clocks may have adapted to different environmental conditions during migrations, consistent with studies on insects [76], birds [77], and fish [78] living at different latitudes. These studies analyzed, in particular, the evolution of circadian genes which may be related to selective pressure exerted from latitude, temperature, ultraviolet radiation flux [79]. Consequently, people living in countries exposed to less than 2000 annual sunshine hours are less susceptible to higher frequency of light during night compared to people living in countries exposed to more annual sunshine hours. An explanation may be the presence of different alleles of their circadian genes, which acted to adapt the organism to different living conditions such as different latitudes. [80]. Finally, we may hypothesize that if people residing in “darkest countries” have artificial light kept on also during the daytime, they may not be as strongly influenced by higher levels of LAN because of different environment-adaptive alleles which acted to adapt the organism to light regimes diverse from the natural ones, as has been shown in animals [81]. In the meantime, another hypothesis that may explain the stronger association in relation to the annual sunshine hours is represented by the cumulative effect of longer daily sunshine hours with LAN exposure. In fact, those living in “brightest countries” are exposed to higher levels of light, which may lead to greater melatonin suppression levels and chronodisruption. Hence, the combination of daily sunshine hours and LAN may increase breast cancer risk.

Our results are relatively consistent with two previous meta-analyses [21, 22], with the exception of the subgroup analysis according to menopausal status, where our results were similar to those of Lai et al. [21] but conflicted with those of Wu et al. [22]. However, to our knowledge this meta-analysis is the first to have assessed the dose–response between LAN and breast cancer risk, particularly among premenopausal women. In addition, owing to three new, recently-published studies we could include in the present review, we could re-assess the LAN-breast cancer relation according to ER cancer type, also performing a dose–response meta-analysis in these subgroups. Though our findings support a harmful effect of LAN in both the ER + and ER − breast cancer subtypes, at high exposure levels i.e., above 30 nW/cm^2^/sr the curve flattened in women with ER + disease but still increased in the ER − subgroup.

Our review has some strengths and limitations. Firstly, we used a newly developed meta-analytic tool for exploring the full shape of the dose–response, enabling us to assess the shape of the relation between LAN exposure and breast cancer risk over a wide range of exposure and across population subgroups. Our approach also yielded some indications of the threshold exposure levels that can increase breast cancer risk. Moreover, we systematically used the most adjusted model from each included study, thereby accounting for major confounders of the association.

Nonetheless, we acknowledge that some summary estimates are still statistically unstable due to the low number of studies still characterizing some subgroups. In addition, we could not rule out that unmeasured confounding was still likely in the investigated studies, and therefore influenced the findings. An example of such potential identified confounder could be the possibility that air pollution is heavier in urban areas, where the highest levels of LAN exposure are also detectable. In particular, LAN exposure may correlate with higher levels of traffic-related pollutants, including noise, as indicated by its inverse correlation with greenness and green space diversity [82, 83]. Unfortunately, only one study included traffic noise in the multivariable model, thus hampering the evaluation of any independent effects of this factor and its potential for confounding in LAN-related studies. Additionally, other confounders may be those related to the occupational night environment, especially for studies assessing LAN exposure among nightshift workers [55]. Another limitation could be the limited capacity of outdoor LAN to adequately reflect personal light exposure due to differences in indoor lightning, use of electronic devices, nighttime activities, or window treatments, being these only some of the potential other sources of exposure [83, 84]. Therefore, future studies should ideally use validated questionnaires combined with satellite data to more accurately measure individual LAN exposure. Finally, funnel plots and trim-and-fill analysis suggest a negligible probability of small-study effects in overall and stratified analyses.

## Conclusions

Our review suggests a positive association between LAN exposure and risk of breast cancer, particularly in some subgroups, especially in premenopausal women, while few differences substantially emerged according to ER status, thus ongoing efforts to minimize LAN exposure might contribute to decrease human burden of diseases [85–87].

## Data Availability

All data generated or analyzed during this study are included in this published article and its additional information files.

## Supple mentary Information

**Additional file 1.** Additional figures and tables.

## Abbreviations

ALAN: Artificial Light At Night
BMI: Body Mass Index
CI: Confidence Interval
ER: Estrogen Receptor
DMSP: US Defense Meteorological Satellite Program Operational Linescan System
DNB: Visible Infrared Imaging Radiometer Suite Day-Night Band
I^2^: Heterogeneity
LAN: Light At Night
RR: Risk Ratio
UV-B: Ultraviolet B
