# Supplementary material for: Light at night and risk of breast cancer: a systematic review and dose–response meta-analysis
Source: Int J Health Geogr. 2021 Oct 16;20:44. doi: 10.1186/s12942-021-00297-7 (PMC8520294; doi:10.1186/s12942-021-00297-7)
Supplement: Supplementary file 1 — Additional file 1. Additional figures and tables. [file 12942_2021_297_MOESM1_ESM.docx]

**Supplemental Table S1***.* Details of search strategy with the search strings used in PubMed, Web of Science, and Embase databases.

| **Database search** | **Search string** | **Results (n)** |
| --- | --- | --- |
| **PubMed** | ("Breast Neoplasms"[Mesh] OR "Breast cancer" [tiab] OR "Breast neoplasm"[tiab]) AND ( "Light/adverse effects"[Mesh] OR "Lighting/adverse effects"[Mesh] OR "Light at night"[tiab]) | 251 |
| **Web of Science** | (TS=("light at night") OR TI=("light at night") OR AB=("light at night")) AND (TS=("breast cancer") OR TI=("breast cancer") OR AB=("breast cancer")) | 262 |
| **Embase** | ('breast cancer'/exp OR 'advanced breast cancer':ti,ab,kw OR 'breast cancer':ti,ab,kw OR 'breast cancer recurrence':ti,ab,kw OR 'breast gland cancer':ti,ab,kw OR 'breast gland neoplasm':ti,ab,kw OR 'cancer, breast':ti,ab,kw OR 'mamma cancer':ti,ab,kw OR 'mammary cancer':ti,ab,kw OR 'mammary gland cancer':ti,ab,kw) AND ('light at':ti,ab OR 'lightning'/exp OR 'lightning':ti,ab,kw OR 'artificial light'/exp) | 291 |

**Supplemental Table S2**. Criteria adopted for Risk of Bias assessment.

| **Domains** | **Criteria** |
| --- | --- |
| Bias due to confounding | Factor considered mandatory to judge a study at moderate risk of bias was age.  Factors considered mandatory to judge a study at low risk of bias are at least two among: hormone replacement therapy use, family history of breast cancer, or smoking. |
| Bias in selecting participants in the study | Selection of eligible participants must not be related to lightning exposure to be at low risk of bias. Otherwise, high risk was considered. |
| Bias in exposure classification | Possible exposure misclassification with moderate risk of bias for studies that relied on self-report for exposure assessment only, high risk of bias if the questionnaire was not validated or self-administrated (i.e. without a trained interviewer). |
| Bias in departure from intended exposure | Studies are considered at low risk of bias if participants are analyzed based on the exposure group they are assigned at the beginning of the study, while are considered at high risk if changing in exposure classification occurred during the study. |
| Bias due to missing data | 10% was considered as reasonable cutpoint for missing data to considered a study at low risk of bias in both case-control and cohort studies. |
| Bias in outcome measurement | Possible bias based on the modality of outcome assessment. High risk in case of assessment based on self-report only, without external validation, moderate with validation. |
| Bias in selection of reported results | For low risk of bias clear evidence that results have not been selected must be present as well as clear and complete reporting of statistical methods. |
| Overall risk of bias | If at least one domain was found at high risk of bias, the overall risk was considered high. If more than one domain was found at moderate risk of bias, the overall risk was considered moderate. If all domains were at low risk of bias, the overall risk was considered low. |

**Supplemental Table S3.** List of all papers excluded with reasons.

| **Full-text articles excluded** | **Reasons** |
| --- | --- |
| **1)** Bauer S, Wagner S, Bayakly R, Vena J: Light-at-night and breast cancer risk in Georgia. Epidemiology 2012, 23(5):S554. **2)** Bukowska A, Sobala W, Peplonska B: Rotating night shift work, sleep quality, selected lifestyle factors and prolactin concentration in nurses and midwives. Chronobiology International 2015, 32(3):318-326. **3)** Datta K, Roy A, Nanda D, Das I, Guha S, Ghosh D, Sikdar S, Biswas J: Association of Breast Cancer with Sleep Pattern - A Pilot Case Control Study in a Regional Cancer Centre in South Asia. Asian Pacific Journal of Cancer Prevention 2014, 15(20):8641-8645. **4)** Schernhammer ES, Laden F, Speizer FE, Willett WC, Hunter DJ, Kawachi I, Colditz GA: Rotating night shifts and risk of breast cancer in women participating in the nurses' health study. J Natl Cancer Inst 2001, 93(20):1563-1568. | Insufficient data (4) |
| **1)** Davis R: Light exposure and breast cancer. Epidemiology 1991, 2(6):458-459. **2)** Haim A, Portnov BA: LAN and breast cancer risk: Can we see a forest through the trees? Response to "measurements of light at night (LAN) for a sample of female school teachers" by M. S. Rea, J. A. Brons, and M. G. Figueiro. Chronobiology International 2011, 28(8):734-736. **3)** Kyba CC, Aronson KJ: Assessing exposure to outdoor lighting and health risks. Epidemiology 2015, 26(4):e50. | Commentary (3) |
| **1)** Reed VA: Shift work, light at night, and the risk of breast cancer. Aaohn j 2011, 59(1):37-45; quiz 46. **2)** Schernhammer E, Schulmeister K: Light at night and cancer risk. Photochemistry and Photobiology 2004, 79(4):316-318. | Review (2) |
| **1)** Wu Y, Gui SY, Fang Y, Zhang M, Hu CY: Exposure to outdoor light at night and risk of breast cancer: A systematic review and meta-analysis of observational studies. Environ Pollut 2021, 269:116114. | Meta-analysis (1) |
| **1)** Al-Naggar RA, Anil S: Artificial Light at Night and Cancer: Global Study. Asian Pac J Cancer Prev 2016, 17(10):4661-4664. | Not pertinent (1) |
| **1)** Kennaway DJ: Light at night, melatonin and breast cancer. Chronobiology international 2014, 31(2):297-298. | Editorial (1) |
| **1)** Kim YJ, Park MS, Lee E, Choi JW: High Incidence of Breast Cancer in Light-Polluted Areas with Spatial Effects in Korea. Asian Pacific journal of cancer prevention: APJCP 2016, 17(1):361-367. | Ecological study (1) |

**Supplemental Table S4***.* Additional characteristics of studies included in the systematic review.

| **First author, year** | **Cancer risk in premenopausal** | | **Cancer risk in postmenopausal** | | **Cancer risk in women BMI <25** | **Cancer risk in women BMI >25** | **Cancer risk in ER + cancers** | **Cancer risk in ER - cancers** | **Cancer risk in black women** | **Cancer risk in white women** |
| --- | --- | --- | --- | --- | --- | --- | --- | --- | --- | --- |
| Bauer 2013 | NA | | NA | | NA | NA | NA | NA | Ref 1.00 | Ref 1.00 |
|  |  |  |  |  |  |  |  |  | 1.04 (0.78-1.38) | 1.07 (0.97-1.16) |
|  |  |  |  |  |  |  |  |  | 1.02 (0.82-1.28) | 1.13 (1.05-1.22) |
| Clarke 2021 |  | |  | |  |  | Ref 1.00 | Ref 1.00 |  |  |
|  |  |  |  |  |  |  | 1.02 (0.83-1.26) | 1.58 (0.92-2.71) |  |  |
|  |  |  |  |  |  |  | 0.88 (0-67-1.14) | 1.56 (0.82-2.98) |  |  |
| Davis 2001 | NA | | NA | | NA | NA | NA | NA | NA | NA |
| Fritschi 2013 | 1.17 (0.94-1.45) | | 1.1 (0.78-1.55) | | NA | NA | NA | NA | NA | NA |
| Garcia-Saenz 2018 | Ref 1.00 | | Ref 1.00 | | NA | NA | Ref 1.00 | Ref 1.00 | NA | NA |
|  | 0.62 (0.32-1.18) | | 0.97 (0.63-1.50) | |  |  | 0.86 (0.6-1.28) | 0.59 (0.2-1.60) |  |  |
|  | 1.09 (0.57-2.09) | | 1.31 (0.84-2.03) | |  |  | 1.26 (0.8-1.88) | 0.64 (0.2-1.85) |  |  |
| Hurley 2014 | Ref 1.00 | | Ref 1.00 | | Ref 1.00 | Ref 1.00 | NA | NA | NA | NA |
|  | 1.20 (0.98-1.48) | | 0.99 (0.88-1.13) | | 1.01 (0.89-1.16) | 1.1 (0.93-1.28) |  |  |  |  |
|  | 1.20 (0.97-1.49) | | 0.99 (0.87-1.13) | | 1.04 (0.91-1.2) | 1.09 (0.92-1.29) |  |  |  |  |
|  | 1.16 (0.93-1.46) | | 1.01 (0.88-1.15) | | 1.05 (0.91-1.21) | 1.08 (0.91-1.28) |  |  |  |  |
|  | 1.34 (1.07-1.69) | | 1.04 (0.90-1.20) | | 1.09 (0.94-1.27) | 1.18 (0.98-1.41) |  |  |  |  |
| James 2017 | Ref 1.00 | | Ref 1.00 | | NA | NA | NA | NA | NA | NA |
|  | 1.02 (0.87-1.19) | | 0.96 (0.80-1.16) | |  |  |  |  |  |  |
|  | 1.08 (0.92-1.26) | | 0.92 (0.77-1.11) | |  |  |  |  |  |  |
|  | 1.12 (0.96-1.31) | | 0.99 (0.82-1.19) | |  |  |  |  |  |  |
|  | 1.2 (1.02-1.41) | | 0.95 (0.78-1.15) | |  |  |  |  |  |  |
| Johns 2018 | Ref 1.00 | | Ref 1.00 | |  |  | Ref 1.00 | Ref 1.00 |  |  |
|  | 0.91 (0.74-1.1) | | 1.05 (0.91-1.22) | |  |  | 1.07 (0.93-1.24) | 0.86 (0.63-1.18) |  |  |
|  | 1.0 (0.81-1.24) | | 1.0 (0.85-1.18) | |  |  | 1.00 (0.86-1.17) | 0.94 (0.67-1.32) |  |  |
| Keshet-Sitton 2016 | NA | | NA | | NA | NA | NA | NA | NA | NA |
| Kloog 2011 | NA | | NA | | NA | NA | NA | NA | NA | NA |
| Li 2010 | Ref 1.00 | | Ref 1.00 | | NA | NA | NA | NA | NA | NA |
|  | 1.1 (0.4-3.6) | | 1.4 (0.7-2.7) | |  |  |  |  |  |  |
|  | Ref 1.00 | | Ref 1.00 | |  |  |  |  |  |  |
|  | 1.0 (0.5-1.8) | | 1.1 (0.8-1.7) | |  |  |  |  |  |  |
|  | Ref 1.00 | | Ref 1.00 | |  |  |  |  |  |  |
|  | 1.1 (0.5-2.5) | | 1.1 (0.6-1.7) | |  |  |  |  |  |  |
| O' Leary 2006 | NA | | NA | | NA | NA | NA | NA | NA | NA |
| Ritonja 2020 | **DNB** | **DMSP** | **DNB** | **DMSP** | NA | NA | NA | NA | NA | NA |
|  | Ref 1.00 | Ref 1.00 | Ref 1.00 | Ref 1.00 |  |  |  |  |  |  |
|  | 1.17 (0.75-1.84) | 1.13 (0.74-1.73) | 0.90 (0.67-1.22) | 1.01 (0.76-1.36) |  |  |  |  |  |  |
|  | 1.06 (0.68-1.67) | 0.85 (0.53-1.34) | 0.90 (0.64-1.27) | 0.82 (0.60-1.14) |  |  |  |  |  |  |
| White 2017 | NA | | NA | | NA | NA | Ref 1.00 | Ref 1.00 | NA | NA |
|  |  |  |  |  |  |  | 1.05 (0.74-1.5) | 1.04 (0.49-2.21) |  |  |
|  |  |  |  |  |  |  | 1.2 (0.97-1.47) | 0.97 (0.61-1.55) |  |  |
|  |  |  |  |  |  |  | 1.11 (0.96-1.3) | 1.01 (0.71-1.43) |  |  |
|  |  |  |  |  |  |  | 1.07 (0.93-1.23) | 0.91 (0.66-1.25) |  |  |
| Xiao 2020 | NA | | Ref 1.00 | | Ref 1.00 | Ref 1.00 | Ref 1.00 | Ref 1.00 | NA | NA |
|  |  |  | 1.01 (0.95-1.07) | | 0.98 (0.84-1.15) | 0.94 (0.78-1.13) | 0.99 (0.91-1.08) | 1.07 (0.89-1.29) |  |  |
|  |  |  | 1.06 (1.00-1.13) | | 1.12 (0.95-1.33) | 0.97 (0.79-1.18) | 1.07 (0.99-1.17) | 1.06 (0.88-1.28) |  |  |
|  |  |  | 1.09 (1.02-1.16) | | 1.13 (0.95-1.35) | 0.96 (0.78-1.18) | 1.08 (0.99-1.17) | 1.06 (0.87-1.29) |  |  |
|  |  |  | 1.10 (1.02-1.18) | | 1.28 (1.05-1.55) | 0.95 (0.76-1.19) | 1.12 (1.02-1.24) | 1.07 (0.85-1.34) |  |  |
| Yang 2019 | Ref 1.00 | | Ref 1.00 | | NA | NA | Ref 1.00 | Ref 1.00 | NA | NA |
|  | 1.1 (0.96-1.88) | | 1.09 (1.00-1.94) | |  |  | 1.1 (0.99-1.76) | 1.08 (1.00-1.91) |  |  |
|  | 1.16 (1.01-2.85) | | 1.14 (1.05-2.46) | |  |  | 1.13 (1.01-2.57) | 1.17 (1.03-2.92) |  |  |
| Xiao 2021 | NA | | NA | | NA | NA | Ref 1.00 | Ref 1.00 | NA | NA |
|  |  |  |  |  |  |  | 0.82 (0.64-1.05) | 1.15 (0.77-1.72) |  |  |
|  |  |  |  |  |  |  | 1.1 (0.86-1.4) | 1.18 (0.78-1.79) |  |  |
|  |  |  |  |  |  |  | 1.02 (0.77-1.34) | 1.09 (0.70-1.69) |  |  |
|  |  |  |  |  |  |  | 1.37 (1.02-1.84) | 1.23 (0.77-1.98) |  |  |

Abbreviations: BMI, body mass index; CI, confidence interval; DMSP, U.S. Defense Meteorological Satellite Program Operational Linescan System; DNB, Visible Infrared Imaging Radiometer Suite Day-Night Band; ER, estrogen receptor; NA: not assessed; NIH–AARP National Institutes of Health–American Association of Retired Persons; Ref, reference; RR, risk ratio.

**Supplemental Table S5**. Summary Risk of Bias (RoB) assessment with overall study-level risk of bias.

| **Studies** | **Design** | **LAN Assessment** | **Bias due to confounding** | **Bias** **in selecting participants in the study** | **Bias in exposure classification** | **Bias in departure from intended exposure** | **Bias due to missing data** | **Bias in outcome measurement** | **Bias in selection of reported results** | **Study-level RoB Judgment** |
| --- | --- | --- | --- | --- | --- | --- | --- | --- | --- | --- |
| (Bauer et al. 2013) | Case-cohort | Outdoor | Moderate | Low | Low | Low | Low | Low | Low | **Moderate** |
| (Clarke et al. 2021) | Cohort | Outdoor | Moderate | Low | Low | Low | Low | Low | Low | **Moderate** |
| (Davis et al. 2001) | Case-control | Indoor | Low | Low | Moderate | Low | Low | Low | Moderate | **Moderate** |
| (Fritschi et al. 2013) | Case-control | Indoor | Moderate | Low | Moderate | Low | Low | Low | Low | **Moderate** |
| (Garcia-Saenz et al. 2018) | Case-cohort | Outdoor and Indoor | Low | Low | Low and Moderate | Low | Low | Low | Low | **Low and Moderate** |
| (Hurley et al. 2014) | Cohort | Outdoor and Indoor | Low | Low | Low and Moderate | Low | Low | Low | Low | **Low and Moderate** |
| (James et al. 2017) | Cohort | Outdoor | Low | Low | Low | Low | Low | Moderate | Low | **Moderate** |
| (Johns et al. 2018) | Case-cohort | Indoor | Low | Low | Moderate | Low | Low | Low | Low | **Moderate** |
| (Keshet-Sitton et al. 2016) | Case-control | Indoor | Moderate | Low | High | Low | Low | Low | Low | **High** |
| (Kloog et al. 2011) | Case-control | Indoor | Low | Low | Moderate | Low | Low | Low | Low | **Moderate** |
| (Li et al. 2010) | Case-control | Indoor | Low | Low | Moderate | Low | Low | Low | Low | **Moderate** |
| (O'Leary et al. 2006) | Case-control | Indoor | Low | Low | Moderate | Low | Moderate | Low | Moderate | **Moderate** |
| (Ritonja et al. 2020) | Case-control | Outdoor | Low | Low | Low | Low | Low | Low | Low | **Low** |
| (White et al. 2017) | Cohort | Indoor | Low | Low | Moderate | Low | Low | Low | Low | **Moderate** |
| (Xiao et al. 2020) | Cohort | Outdoor | Low | Low | Low | Low | Low | Low | Low | **Low** |
| (Xiao et al. 2021) | Cohort | Outdoor | Low | Low | Low | Low | Low | Low | Low | **Low** |
| (Yang et al. 2019) | Case-control | Indoor | Low | Low | Moderate | Low | Low | Low | Low | **Moderate** |

**Supplemental Table S6.** Summary risk ratios (RRs) and 95% confidence interval (CI) for the association between breast cancer risk and light at night exposure comparing the highest versus the lowest exposure categories for overall study population and selected subgroups, excluding the study we considered at high risk of bias (Keshet-Sitton et al. 2016).

|  | **ALL** | | | **OUTDOOR** | | | **INDOOR** | | |
| --- | --- | --- | --- | --- | --- | --- | --- | --- | --- |
| *Breast cancer* | ***n*** | **RR (95% CI)** | ***I^2^* (%)** | ***n*** | **RR (95% CI)** | ***I^2^* (%)** | ***n*** | **RR (95% CI)** | ***I^2^* (%)** |
| **All women** | 16 | 1.10 (1.06-1.14) | 0.0 | 8 | 1.11 (1.07-1.16) | 0.0 | 8 | 1.06 (0.98-1.14) | 0.0 |
| **Study design** |  |  |  |  |  |  |  |  |  |
| Cohort/case-cohort studies | 9 | 1.11 (1.07-1.15) | 0.0 | 8 | 1.11 (1.07-1.16) |  | 4 | 1.05 (0.96-1.15) | 0.0 |
| Case-control studies | 7 | 1.06 (0.93-1.20) | 11.0 | 1 | 0.95 (0.71-1.28) | - | 6 | 1.08 (0.94-1.25) | 17.1 |
| **Menopausal status** |  |  |  |  |  |  |  |  |  |
| Premenopausal | 8 | 1.16 (1.04-1.28) | 2.3 | 4 | 1.22 (1.08-1.39) | 0.0 | 4 | 1.04 (0.88-1.23) | 0.0 |
| Postmenopausal | 9 | 1.07 (1.02-1.13) | 0.0 | 5 | 1.07 (1.00-1.14) | 5.9 | 4 | 1.08 (0.95-1.23) | 4.3 |
| **BMI** |  |  |  |  |  |  |  |  |  |
| <25 kg/m2 | 2 | 1.17 (1.00-1.36) | 38.9 | 2 | 1.17 (1.00-1.36) | 39.0 | - | - | - |
| ≥25 kg/m2 | 2 | 1.07 (0.87-1.32) | 53.8 | 2 | 1.07 (0.87-1.32) | 53.8 | - | - | - |
| **Estrogen receptor status** |  |  |  |  |  |  |  |  |  |
| ER + | 7 | 1.09 (1.02-1.17) | 0.0 | 4 | 1.12 (0.95-1.32) | 44.8 | 3 | 1.06 (0.95-1.18) | 0.0 |
| ER - | 7 | 1.07 (0.92-1.23) | 0.0 | 4 | 1.12 (0.92-1.35) | 0.0 | 3 | 1.01 (0.81-1.25) | 0.0 |
| **LAN/sunshine hours** |  |  |  |  |  |  |  |  |  |
| < 2000 h. | 3 | 0.99 (0.89-1.11) | 0.0 | 2 | 0.96 (0.80-1.16) | 0.0 | 1 | 1.01 (0.88-1.15) | - |
| 2000-3000 h. | 10 | 1.12 (1.08-1.17) | 0.0 | 5 | 1.12 (1.07-1.17) | 0.0 | 7 | 1.13 (1.02-1.25) | 0.0 |
| > 3000 h. | 2 | 1.08 (0.79-1.47) | 0 | 1 | 1.47 (1.00-2.17) | - | 2 | 0.93 (0.75-1.14) | 0.0 |
| **Equinoctial UVB** |  |  |  |  |  |  |  |  |  |
| 0.0-0.58 | 3 | 0.97 (0.87-1.08) | 0.0 | 2 | 0.91 (0.76-1.09) | 0.0 | 1 | 1.01 (0.88-1.15) | - |
| 0.58-1.15 | 1 | 1.27 (0.89-1.82) | - | 1 | 1.47 (1.00-2.17) | - | 1 | 1.01 (0.60-1.70) | - |
| 1.15-1.73 | 12 | 1.14 (1.10-1.18) | 4.7 | 5 | 1.12 (1.07-1.17) | 0.0 | 8 | 1.18 (1.11-1.26) | 0.7 |

**Supplemental Table S7.** Summary risk ratios (RRs) and 95% confidence interval (CI) for the association between breast cancer risk and light at night exposure comparing the highest versus the lowest exposure categories for overall study population and selected subgroups, excluding the study we considered at high risk of bias (Keshet-Sitton et al. 2016) and the two at moderate risk of bias in selection of reported results (Davis et al. 2001; O'Leary et al. 2006).

|  | **ALL** | | | **OUTDOOR** | | | **INDOOR** | | |
| --- | --- | --- | --- | --- | --- | --- | --- | --- | --- |
| *Breast cancer* | ***n*** | **RR (95% CI)** | ***I^2^* (%)** | ***n*** | **RR (95% CI)** | ***I^2^* (%)** | ***n*** | **RR (95% CI)** | ***I^2^* (%)** |
| **All women** | 14 | 1.10 (1.06-1.15) | 0.0 | 7 | 1.11 (1.07-1.16) | 0.0 | 8 | 1.06 (0.98-1.15) | 0.0 |
| **Study design** |  |  |  |  |  |  |  |  |  |
| Cohort/case-cohort studies | 9 | 1.11 (1.07-1.15) | 0.0 | 8 | 1.11 (1.07-1.16) |  | 4 | 1.05 (0.96-1.15) | 0.0 |
| Case-control studies | 5 | 1.07 (0.90-1.26) | 28.9 | 1 | 0.95 (0.71-1.28) | - | 4 | 1.11 (0.89-1.37) | 38.3 |
| **Menopausal status** |  |  |  |  |  |  |  |  |  |
| Premenopausal | 8 | 1.16 (1.04-1.28) | 2.3 | 4 | 1.22 (1.08-1.39) | 0.0 | 4 | 1.04 (0.88-1.23) | 0.0 |
| Postmenopausal | 9 | 1.07 (1.02-1.13) | 0.0 | 5 | 1.07 (1.00-1.14) | 5.9 | 4 | 1.08 (0.95-1.23) | 4.3 |
| **BMI** |  |  |  |  |  |  |  |  |  |
| <25 kg/m2 | 2 | 1.17 (1.00-1.36) | 38.9 | 2 | 1.17 (1.00-1.36) | 39.0 | - | - | - |
| ≥25 kg/m2 | 2 | 1.07 (0.87-1.32) | 53.8 | 2 | 1.07 (0.87-1.32) | 53.8 | - | - | - |
| **Estrogen receptor status** |  |  |  |  |  |  |  |  |  |
| ER + | 7 | 1.09 (1.02-1.17) | 0.0 | 4 | 1.12 (0.95-1.32) | 44.8 | 3 | 1.06 (0.95-1.18) | 0.0 |
| ER - | 7 | 1.07 (0.92-1.23) | 0.0 | 4 | 1.12 (0.89-1.35) | 0.0 | 3 | 1.01 (0.81-1.25) | 0.0 |
| **LAN/sunshine hours** |  |  |  |  |  |  |  |  |  |
| < 2000 h. | 3 | 0.97 (0.87-1.08) | 0.0 | 2 | 0.96 (0.80-1.16) | 0.0 | 1 | 1.01 (0.88-1.15) | - |
| 2000-3000 h. | 9 | 1.12 (1.08-1.17) | 0.0 | 5 | 1.12 (1.07-1.17) | 0.0 | 5 | 1.14 (1.02-1.28) | 0.0 |
| > 3000 h. | 2 | 1.08 (0.79-1.47) | 0.0 | 1 | 1.47 (1.00-2.17) | - | 2 | 0.93 (0.75-1.14) | 0.0 |
| **Equinoctial UVB** |  |  |  |  |  |  |  |  |  |
| 0.0-0.58 | 3 | 0.97 (0.87-1.08) | 0.0 | 2 | 0.91 (0.76-1.09) | 0.0 | 1 | 1.01 (0.88-1.15) | - |
| 0.58-1.15 | 1 | 1.27 (0.89-1.82) | - | 1 | 1.47 (1.00-2.17) | - | 1 | 1.01 (0.60-1.70) | - |
| 1.15-1.73 | 10 | 1.14 (1.10-1.19) | 4.7 | 5 | 1.12 (1.07-1.17) | 0.0 | 6 | 1.19 (1.12-1.27) | 0.0 |

**Supplemental Table S8.** Summary risk ratios (RRs) and 95% confidence interval (CI) for the association between breast cancer risk and light at night exposure comparing the highest versus the lowest exposure categories for overall study population and selected subgroups, excluding the study we considered at high risk of bias (Keshet-Sitton et al. 2016), the two at moderate risk of bias in selection of reported results (Davis et al. 2001; O'Leary et al. 2006) and the studies at moderate risk of bias due to confounding (Bauer et al. 2013; Clarke et al. 2021; Fritschi et al. 2013; Keshet-Sitton et al. 2016).

|  | **ALL** | | | **OUTDOOR** | | | **INDOOR** | | |
| --- | --- | --- | --- | --- | --- | --- | --- | --- | --- |
| *Breast cancer* | ***n*** | **RR (95% CI)** | ***I^2^* (%)** | ***n*** | **RR (95% CI)** | ***I^2^* (%)** | ***n*** | **RR (95% CI)** | ***I^2^* (%)** |
| **All women** | 11 | 1.10 (1.06-1.15) | 0.0 | 6 | 1.12 (1.06-1.18) | 0.0 | 7 | 1.04 (0.96-1.13) | 0.0 |
| **Study design** |  |  |  |  |  |  |  |  |  |
| Cohort/case-cohort studies | 7 | 1.11 (1.06-1.16) | 0.0 | 6 | 1.12 (1.06-1.18) | 0.0 | 4 | 1.05 (0.96-1.15) | 0.0 |
| Case-control studies | 4 | 0.98 (0.83-1.15) | 0.0 | 1 | 0.95 (0.71-1.28) | - | 3 | 1.02 (0.80-1.31) | 19.8 |
| **Menopausal status** |  |  |  |  |  |  |  |  |  |
| Premenopausal | 7 | 1.16 (1.03-1.30) | 2.3 | 4 | 1.22 (1.08-1.39) | 0.0 | 3 | 1.02 (0.84-1.24) | 0.0 |
| Postmenopausal | 8 | 1.06 (1.00-1.13) | 0.0 | 5 | 1.07 (1.00-1.14) | 5.9 | 3 | 1.03 (0.89-1.20) | 0.0 |
| **BMI** |  |  |  |  |  |  |  |  |  |
| <25 kg/m2 | 2 | 1.17 (1.00-1.36) | 38.9 | 2 | 1.17 (1.00-1.36) | 39.0 | - | - | - |
| ≥25 kg/m2 | 2 | 1.07 (0.87-1.32) | 53.8 | 2 | 1.07 (0.87-1.32) | 53.8 | - | - | - |
| **Estrogen receptor status** |  |  |  |  |  |  |  |  |  |
| ER + | 7 | 1.09 (1.02-1.17) | 0.0 | 4 | 1.12 (0.95-1.32) | 44.8 | 3 | 1.06 (0.95-1.18) | 0.0 |
| ER - | 7 | 1.07 (0.92-1.23) | 0.0 | 4 | 1.12 (0.89-1.35) | 0.0 | 3 | 1.01 (0.81-1.25) | 0.0 |
| **LAN/sunshine hours** |  |  |  |  |  |  |  |  |  |
| < 2000 h. | 2 | 1.00 (0.88-1.13) | 0.0 | 1 | 0.95 (0.71-1.21) | 0.0 | 1 | 1.01 (0.88-1.15) | - |
| 2000-3000 h. | 7 | 1.12 (1.07-1.18) | 0.0 | 4 | 1.12 (1.06-1.18) | 0.0 | 4 | 1.11 (0.98-1.27) | 0.0 |
| > 3000 h. | 2 | 1.08 (0.79-1.47) | 0.0 | 1 | 1.47 (1.00-2.17) | - | 2 | 0.93 (0.75-1.14) | 0.0 |
| **Equinoctial UVB** |  |  |  |  |  |  |  |  |  |
| 0.0-0.58 | 2 | 0.95 (0.80-1.14) | 37.1 | 1 | 0.83 (0.63-1.09) | - | 1 | 1.01 (0.88-1.15) | - |
| 0.58-1.15 | 1 | 1.27 (0.89-1.82) | - | 1 | 1.47 (1.00-2.17) | - | 1 | 1.01 (0.60-1.70) | - |
| 1.15-1.73 | 8 | 1.15 (1.09-1.20) | 11.7 | 4 | 1.12 (1.06-1.18) | 0.0 | 5 | 1.18 (1.10-1.28) | 6.1 |

**Supplemental Figure S1.** Risk ratio (RR) with 95% confidence interval (CI) for the association between light at night exposure and risk of breast (N=17 studies) comparing the highest versus the lowest exposure category in cohort/case-cohort and case-control studies. The squares represent point estimates of RR and horizontal lines represent their 95% confidence intervals (CIs). The area of each square is proportional to the inverse of the variance of the estimated log RR. The diamonds represent the combined RR for each subgroup and the overall RR for all studies. The solid line represents RR=1.

**Supplemental Figure S2.** Risk ratio (RR) with 95% confidence interval (CI) for the association between light at night exposure and risk of breast in premenopausal and postmenopausal, divided in cohort/case-cohort (A, N=5 studies) and case-control (B, N=4 studies). The analysis compares the highest versus the lowest exposure category. The area of each grey square is proportional to the inverse of the variance of the estimated log RR. Black diamonds represent point estimates of RR and horizontal lines represent their 95% confidence intervals (CIs). The open diamonds represent the combined RR for each subgroup and the overall RR for all studies. The solid line represents RR=1.

**Supplemental Figure S3.** Risk ratio (RR) with 95% confidence interval (CI) for the association between light at night exposure and risk of breast (N=7 studies) according to ER (estrogen receptor) status, comparing the highest versus the lowest exposure category. The area of each grey square is proportional to the inverse of the variance of the estimated log RR. Black diamonds represent point estimates of RR and horizontal lines represent their 95% confidence intervals (CIs). The open diamonds represent the combined RR for each subgroup and the overall RR for all studies. The solid line represents RR=1.

**Supplemental Figure S4.** Dose-response meta-analysis between light at night and risk of breast cancer in women with ER- and ER+ breast cancer (Xiao et al. 2020; Xiao et al. 2021; Clarke et al. 2021).Spline curve (black solid line) with 95% confidence limits (black dashed lines), and linear trend (long-dashed gray line). RR, risk ratio.

**
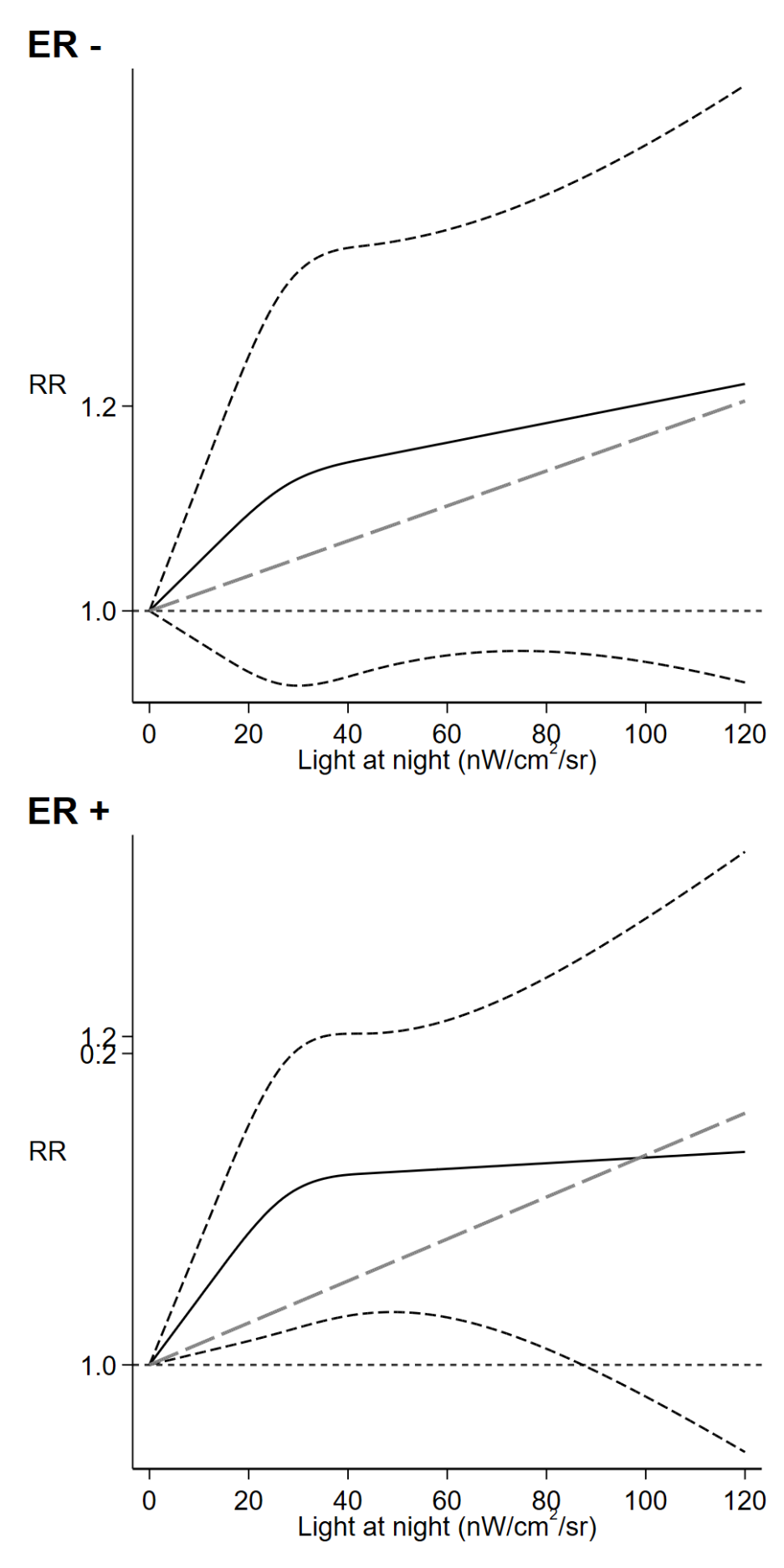
**

**Supplemental Figure S5.** Risk ratio (RR) with 95% confidence interval (CI) for the association between light at night exposure and risk of breast (N=2 studies) in women with BMI<25 and women with BMI>25, comparing the highest versus the lowest exposure category. The area of each grey square is proportional to the inverse of the variance of the estimated log RR. Black diamonds represent point estimates of RR and horizontal lines represent their 95% confidence intervals (CIs). The open diamonds represent the combined RR for each subgroup and the overall RR for all studies. The solid line represents RR=1.

**Supplemental Figure S6.** Dose-response meta-analysis between light at night and risk of breast cancer in women with a BMI (body mass index) <25 (**A**) and women with a BMI≥25 (**B**) (Hurley et al. 2014; Xiao et al. 2020). Spline curve (black solid line) with 95% confidence limits (black dashed lines), and linear trend (long-dashed gray line). RR, risk ratio.


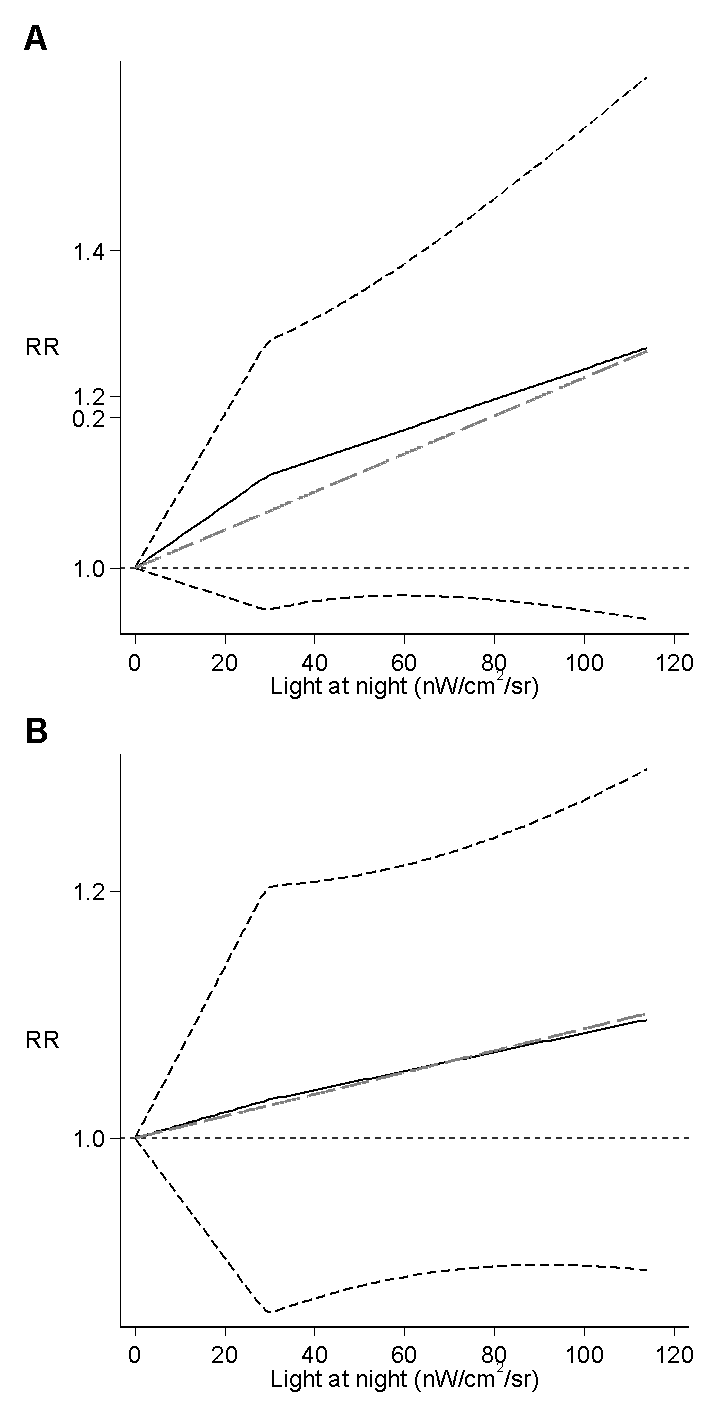


**Supplemental Figure S7**. Annual sunshine hours map of the world. Dark blue indicates countries receiving less than 1200 hours of annual sunshine; blue shows countries with 1200-1600 sunshine hours; light blue indicates countries receiving 1600-2000 sunshine hours; dark green refers to countries with 2000-2400 sunshine hours; light green shows countries receiving 2400-3000 hours of sunshine per year; yellow indicates countries with 3000-3600 sunshine hours; orange refers to countries receiving 3600-4000 sunshine hours; red shows countries with more than 4000 annual sunshine hours (De Jong et al. 2017).


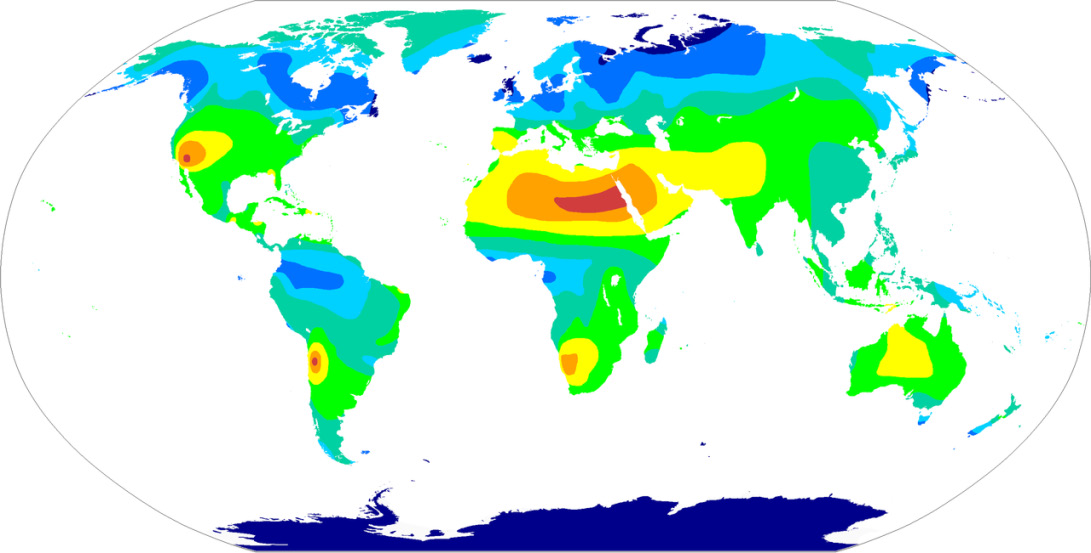


**Supplemental Figure S8.** Risk ratio (RR) with 95% confidence interval (CI) for the association between light at night exposure and risk of breast (N=17 studies) according to annual sunshine hours exposure.

**Supplemental Figure S9** Risk ratio (RR) with 95 % confidence interval (CI) for the association between light at night exposure and risk of breast according to annual sunshine hours exposure (A: <2000h; B: 2000-3000h; C: >3000h) divided by menopausal status.

**Supplemental Figure S10**. Risk ratio (RR) with 95 % confidence interval (CI) for the association between light at night exposure and risk of breast according to annual sunshine hours (A: <2000h; B: 2000-3000h; C: >3000h) divided by outdoor or indoor exposure.

**Supplemental Figure S11.** Estimated equinoctial ultraviolet B flux (280-315 nm) at ground level, solar noon. Unit of measurement: Watts/meter^2^ (Mohr et al. 2005).

**
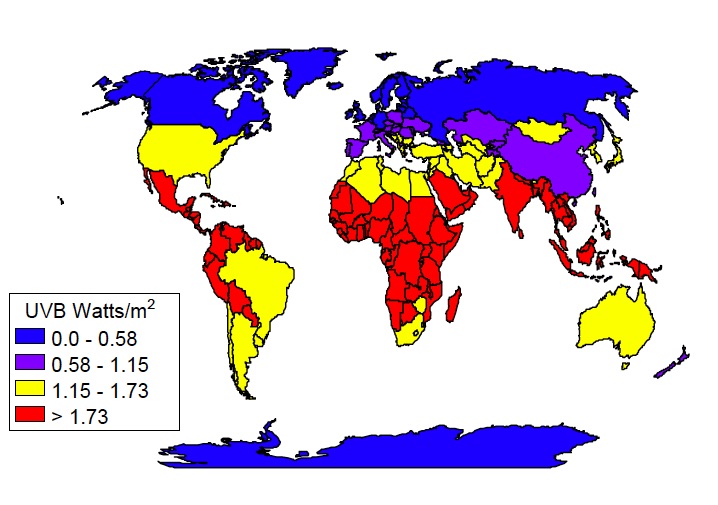
**

**Supplemental Figure S12.** Risk ratio (RR) with 95 % confidence interval (CI) for the association between light at night exposure and risk of breast according to estimated equinoctial ultraviolet B irradiance (280-315 nm) at ground level, solar noon.

^^

**Supplemental Figure S13.** Dose-response meta-analysis (with sensitivity analysis) between light at night and risk of breast cancer in all women (Bauer et al. 2013; Hurley et al. 2014; James et al. 2017; Ritonja et al. 2020; Xiao et al. 2020; Clarke et al. 2021) and between light at night and risk of breast cancer in premenopausal (Hurley et al. 2014; James et al. 2017; Ritonja et al. 2020) and postmenopausal women (Hurley et al. 2014; James et al. 2017; Ritonja et al. 2020; Xiao et al. 2020). Spline curve (black solid line) with 95% confidence limits (black dashed lines), and linear trend (long-dashed gray line). Light grey lines indicate confidence interval for sensitivity analysis. RR, risk ratio.

**Supplemental Figure S14.** Funnel plot of small-study bias assessment for breast cancer risk. Black diamonds represent studies included in each analysis, the x-axis indicates the study effect through its risk ratio (RR), and the y-axis indicates study precision through its standard error. The outer dashed lines indicate the triangular region within which 95% of studies are expected to lie in the absence of both biases and heterogeneity. The solid vertical line corresponds to overall summary RR for meta-analysis of included studies.

**Supplemental Figure S15.** Funnel plot of small-study bias assessment for breast cancer risk according to menopausal status. Black diamonds represent studies included in each analysis, the x-axis indicates the study effect through its risk ratio (RR), and the y-axis indicates study precision through its standard error. The outer dashed lines indicate the triangular region within which 95% of studies are expected to lie in the absence of both biases and heterogeneity. The solid vertical line corresponds to overall summary RR for meta-analysis of included studies.

**Supplemental Figure S16.** Funnel plot Funnel plot of small-study bias assessment for breast cancer risk according to exposure assessment method. Black diamonds represent studies included in each analysis, the x-axis indicates the study effect through its risk ratio (RR), and the y-axis indicates study precision through its standard error. The outer dashed lines indicate the triangular region within which 95% of studies are expected to lie in the absence of both biases and heterogeneity. The solid vertical line corresponds to overall summary RR for meta-analysis of included studies.

**References for supplemental material**

Bauer, S. E., S. E. Wagner, J. Burch, R. Bayakly, and J. E. Vena. 2013. 'A case-referent study: light at night and breast cancer risk in Georgia', *Int J Health Geogr*, 12: 23.

Clarke, R. B., H. Amini, P. James, M. von Euler-Chelpin, J. T. Jorgensen, A. Mehta, T. Cole-Hunter, R. Westendorp, L. H. Mortensen, S. Loft, J. Brandt, O. Hertel, M. Ketzel, C. Backalarz, Z. J. Andersen, and Y. H. Lim. 2021. 'Outdoor light at night and breast cancer incidence in the Danish Nurse Cohort', *Environ Res*, 194: 110631.

Davis, S., D. K. Mirick, and R. G. Stevens. 2001. 'Night shift work, light at night, and risk of breast cancer', *J Natl Cancer Inst*, 93: 1557-62.

De Jong, S , W. L. Auping, W.T. Oosterveld, A. Usanov, M. Abdalla, A. Van de Bovenkamp, and C. Frattina. 2017. 'The geopolitical impact of climate mitigation policies: How hydrocarbon exporting rentier states and developing nations can prepare for a more sustainable future', *The Hague Centre for Strategic Studies*.

Fritschi, L., T. C. Erren, D. C. Glass, J. Girschik, A. K. Thomson, C. Saunders, T. Boyle, S. El-Zaemey, P. Rogers, S. Peters, T. Slevin, A. D'Orsogna, F. de Vocht, R. Vermeulen, and J. S. Heyworth. 2013. 'The association between different night shiftwork factors and breast cancer: a case-control study', *Br J Cancer*, 109: 2472-80.

Hurley, S., D. Goldberg, D. Nelson, A. Hertz, P. L. Horn-Ross, L. Bernstein, and P. Reynolds. 2014. 'Light at night and breast cancer risk among California teachers', *Epidemiology*, 25: 697-706.

James, P., K. A. Bertrand, J. E. Hart, E. S. Schernhammer, R. M. Tamimi, and F. Laden. 2017. 'Outdoor light at night and breast cancer incidence in the Nurses' Health Study II', *Environ Health Perspect*, 125: 087010.

Keshet-Sitton, A., K. Or-Chen, S. Yitzhak, I. Tzabary, and A. Haim. 2016. 'Can avoiding light at night reduce the risk of breast cancer?', *Integr Cancer Ther*, 15: 145-52.

Mohr, Sharif, Cedric Garland, Edward Gorham, William Grant, Robyn Highfill, and Frank Garland. 2005. 'Mapping Vitamin D deficiency, breast cancer, and colorectal cancer'.

O'Leary, E. S., E. R. Schoenfeld, R. G. Stevens, G. C. Kabat, K. Henderson, R. Grimson, M. D. Gammon, M. C. Leske, Fields Electromagnetic, and Group Breast Cancer on Long Island Study. 2006. 'Shift work, light at night, and breast cancer on Long Island, New York', *Am J Epidemiol*, 164: 358-66.

Ritonja, J., M. A. McIsaac, E. Sanders, C. C. M. Kyba, A. Grundy, E. Cordina-Duverger, J. J. Spinelli, and K. J. Aronson. 2020. 'Outdoor light at night at residences and breast cancer risk in Canada', *Eur J Epidemiol*, 35: 579-89.

Xiao, Q., G. L. Gierach, C. Bauer, W. J. Blot, P. James, and R. R. Jones. 2021. 'The Association between Outdoor Artificial Light at Night and Breast Cancer Risk in Black and White Women in the Southern Community Cohort Study', *Environ Health Perspect*, 129: 87701.

Xiao, Q., P. James, P. Breheny, P. Jia, Y. Park, D. Zhang, J. A. Fisher, M. H. Ward, and R. R. Jones. 2020. 'Outdoor light at night and postmenopausal breast cancer risk in the NIH-AARP diet and health study', *Int J Cancer*, 147: 2363-72.
